# Compartment-Specific Transcriptional Remodelling Defines Denervation and Geriatric Muscle Regeneration

**DOI:** 10.3390/ijms27209025

**Published:** 2026-10-12

**Authors:** Wanting Tong, Qi Yu, Renyu Yang, Chunxue Shao, Guang Yang, Ziheng Wang

**Affiliations:** Division of Computational Biology, Chinese Center of Exercise Epidemiology, Northeast Normal University, Changchun 130024, China

**Keywords:** skeletal muscle, ageing, denervation, regeneration, single-cell RNA sequencing, pseudobulk, neuromuscular junction

## Abstract

Neuromuscular decline involves motor, junctional, myofibre and niche dysfunction, but whether these components remodel as a coordinated system is unresolved. We harmonised 654,053 locally audited single-cell or single-nucleus profiles from two human ageing cohorts and four mouse studies spanning denervation, injury, reinnervation and exercise, retaining the donor, animal or library as the inferential unit. Denervation produced 4311 cell-type-specific differential-expression results, with the largest number of detected gene effects in type IIB myonuclei. Competitive animal-pseudobulk testing identified eight FDR-supported compartment–programme results; six denervation-sensitivity results remained supported after each single-gene deletion, whereas the FAP matrix-inflammatory and type IIB junctional results were gene-sensitive. During geriatric regeneration, all eight annotated broad compartments varied over time, and 26 of 34 analysis-defined biologically applicable compartment–programme tests were significant after FDR correction. Tests of human ageing, restricted age-by-time interactions and mitochondrial perturbation did not survive multiplicity correction. The human results are hypothesis-generating, and neither RNA scores nor predicted cell communication establish functional recovery. This resource supports compartment-specific transcriptional responses within denervation and geriatric injury designs; it does not establish a conserved recovery axis across the six contexts.

## 1. Introduction

Skeletal-muscle function emerges from a distributed neuromuscular system. Motor axons determine activation; the neuromuscular junction (NMJ) translates neural input into myofibre depolarisation; specialised myonuclei maintain synaptic and contractile domains; and endothelial, immune, Schwann and mesenchymal populations sustain perfusion, trophic support and repair. Ageing, inactivity and nerve injury can disturb each layer, with motor-unit loss, denervation–reinnervation cycles and junctional instability contributing to sarcopenic decline [1,2,3,4,5,6,7]. Their effects need not be synchronous. A muscle may recover mass without restoring motor-unit organisation, or resolve inflammation while energetic and junctional programmes remain abnormal. Understanding decline and recovery therefore requires measurement across cell compartments rather than reduction to a single myofibre-centred endpoint.

Single-cell and single-nucleus atlases have transformed this problem. Human atlases resolve age-associated remodelling of myonuclei and the surrounding niche, including rare NMJ-associated populations that are largely inaccessible to bulk profiling [8,9]. Myofibre studies further show that multinucleated syncytia contain spatially and transcriptionally specialised nuclear domains [10,11,12]. These observations make a compartmental model of neuromuscular ageing biologically plausible. They do not, however, establish whether age-associated states are induced by denervation, reversed during regeneration or coordinated with reinnervation. Cross-sectional human biopsies offer disease relevance but limited temporal resolution; mouse perturbations provide temporal and mechanistic contrasts but only partial correspondence to chronic human ageing.

Regeneration adds another layer of complexity. Pax7-positive satellite cells are required for efficient acute injury repair, but their competence declines with advanced age, and both systemic and local niche signals can restrict or restore regenerative potential [13,14,15,16,17,18,19,20,21]. FAPs, macrophages, regulatory lymphocytes and endothelial cells form a dynamic niche whose inflammatory, matrix and vascular functions must be engaged and then resolved [22,23,24,25,26,27,28,29]. This multicellular biology is already established: FAPs support myogenesis while also producing matrix, immune populations influence myogenic progression and stromal fate, and endothelial cells interact with the satellite-cell niche. These studies motivate compartment-aware analysis; identifying these populations or their co-occurring responses is not itself evidence of a new communication mechanism. Denervation, by contrast, initiates a transcriptional programme in myofibres while simultaneously altering synaptic, stromal and immune states. Reinnervation must then restore a postsynaptic apparatus and its local energetic support, processes that become less reliable with ageing [30,31]. The central unresolved question is therefore not simply which genes change, but whether the constituent programmes of the neuromuscular system change together, and whether any apparent recovery axis generalises across perturbations, ages and species.

This question is also statistical. Thousands of cells from one donor or animal do not constitute thousands of independent experimental units. Cell-level testing can substantially inflate significance when within-subject correlation is ignored, whereas replicate-aware pseudobulk approaches generally provide better false-discovery control [32,33,34,35]. Public studies further differ in sampling depth, capture modality, time-point balance and availability of biological replicates. Combining all cells into one integrated embedding may aid annotation, but it cannot turn cross-sectional donors, destructive mouse time courses and condition-level aggregates into a single longitudinal experiment. A credible cross-study analysis must consequently couple every claim to its biological replicate, estimable contrast and multiplicity family.

Here, we assembled an evidence-gated resource spanning two human ageing cohorts and four mouse studies of denervation, injury-induced regeneration, nerve-crush reinnervation with mitochondrial perturbation and exercise. The contribution is a transparent comparative resource and a replicate-aware reanalysis of established biological settings, rather than a new statistical method or demonstration of a novel signalling mechanism. We analysed 654,053 locally audited profiles while retaining the original inferential units, compartment-appropriate candidate programmes and null or unavailable tests. Our specific aims were: (i) to identify the compartments and candidate programmes most responsive to replicated denervation; (ii) to describe composition and programme variation across a six-time-point geriatric injury series and separately estimate the restricted young-versus-geriatric interaction at the two shared days; and (iii) to assess the evidence and limitations for corresponding programme associations in human ageing, a reinnervation perturbation and descriptive exercise aggregates. The strongest evidence concerns denervation and temporal transcriptional remodelling in geriatric muscle. Human ageing and restricted interaction tests do not provide FDR-supported validation, and a non-significant perturbation interaction does not establish absence of a biological effect. The comparison therefore evaluates the limits of a shared recovery hypothesis without claiming to prove or disprove universal neuromuscular coordination.

## 2. Results

### 2.1. Six-Cohort Coverage and Cohort-Specific Statistical Units

**Figure 1:**
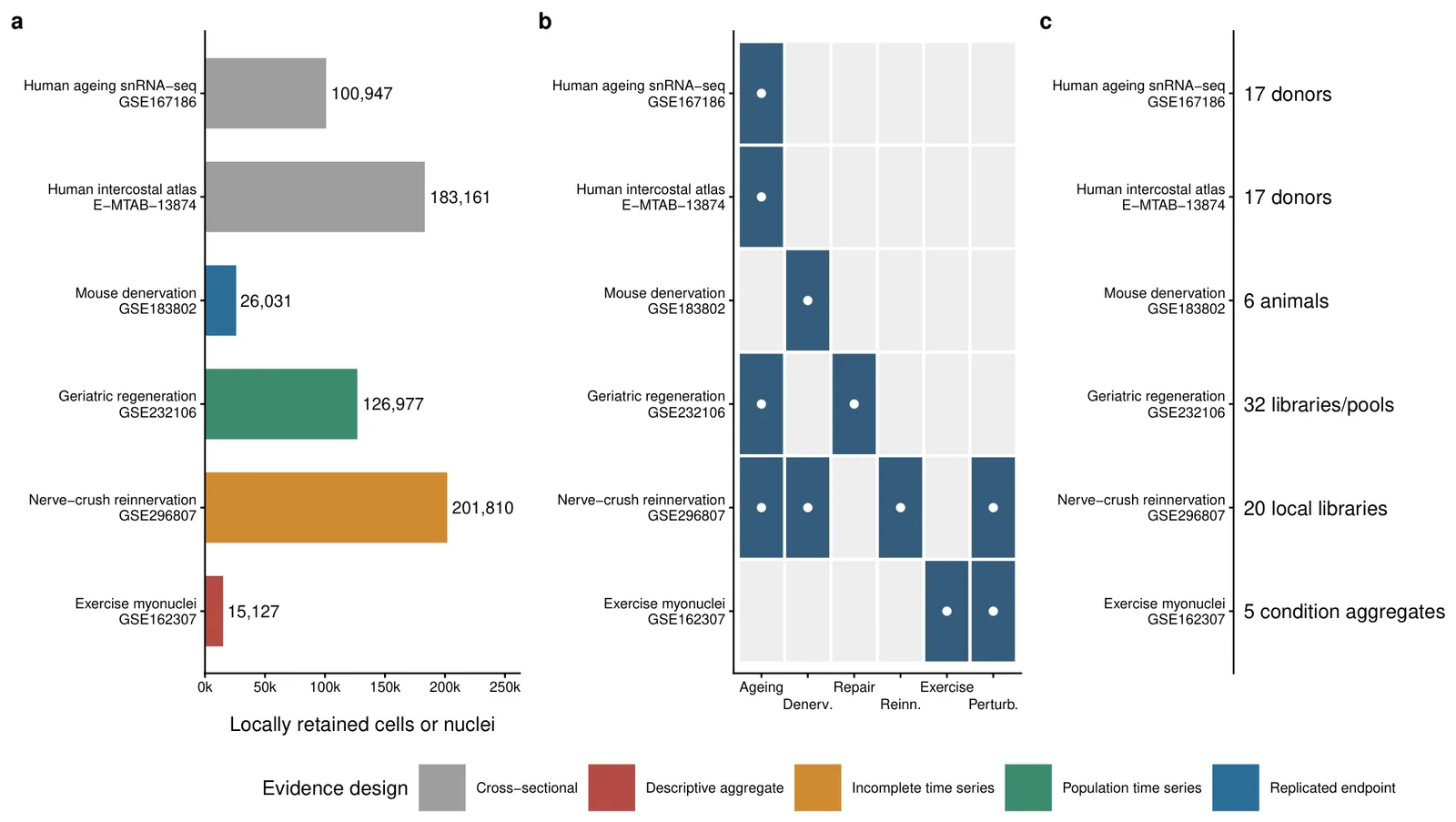
Study architecture and evidence tiers. Six public single-cell or single-nucleus datasets were audited before integration. (**a**) Locally retained cell or nucleus counts for each cohort; bar colors indicate evidence design (cross-sectional, replicated endpoint, population time series, incomplete time series or descriptive aggregate). (**b**) Study-design coverage across ageing, denervation, repair, reinnervation, exercise and perturbation; dots indicate represented cohort–context combinations. (**c**) Statistical units for each dataset: 17 donors per human cohort, 6 animals for denervation, 32 libraries or pools for geriatric regeneration, 20 local libraries for nerve-crush reinnervation and 5 condition aggregates for exercise. Evidence tiers reflect biological replication, availability of the relevant factorial cells and whether analyses are confirmatory or descriptive. Counts refer to the conservative primary analysis branch. Missing multiplexed young libraries in GSE296807 and the lack of replicate-level processed exercise profiles are shown explicitly.

We downloaded and locally audited six public datasets comprising two human and four mouse studies (Figure 1; Table 1; Supplementary Table S1). The conservative primary branch retained 654,053 cells or nuclei: 100,947 from GSE167186, 183,161 from E-MTAB-13874, 26,031 from GSE183802, 126,977 from the locally available GSE232106 subset, 201,810 from GSE296807 and 15,127 from GSE162307. The GSE232106 main branch used fixed expression thresholds consistent with the source analysis; an adaptive quality-control sensitivity retained 140,084 cells. Each retained profile was linked to its source accession, sample and processing branch.

The studies differed substantially in inferential resolution. The statistical units were 17 donors in each human cohort, 6 animals in the denervation experiment, 32 libraries or pools in the local regeneration subset, 20 locally available nerve-crush matrices and 5 processed exercise aggregates. We therefore treated resource scale as a measure of cellular coverage, not replication. This audit also identified two claim-limiting gaps. First, six young animals in GSE296807 were available only through three multiplexed gene-expression libraries and three paired cell-multiplexing libraries in the Sequence Read Archive, preventing a balanced local age-by-time analysis. Second, GSE162307 supplied one processed aggregate per public condition, precluding replicate-based inference. These limitations were encoded in the evidence ledger rather than resolved through imputation.

### 2.2. Denervation Response Is Centred on Fast-Glycolytic Myonuclei

**Figure 2:**
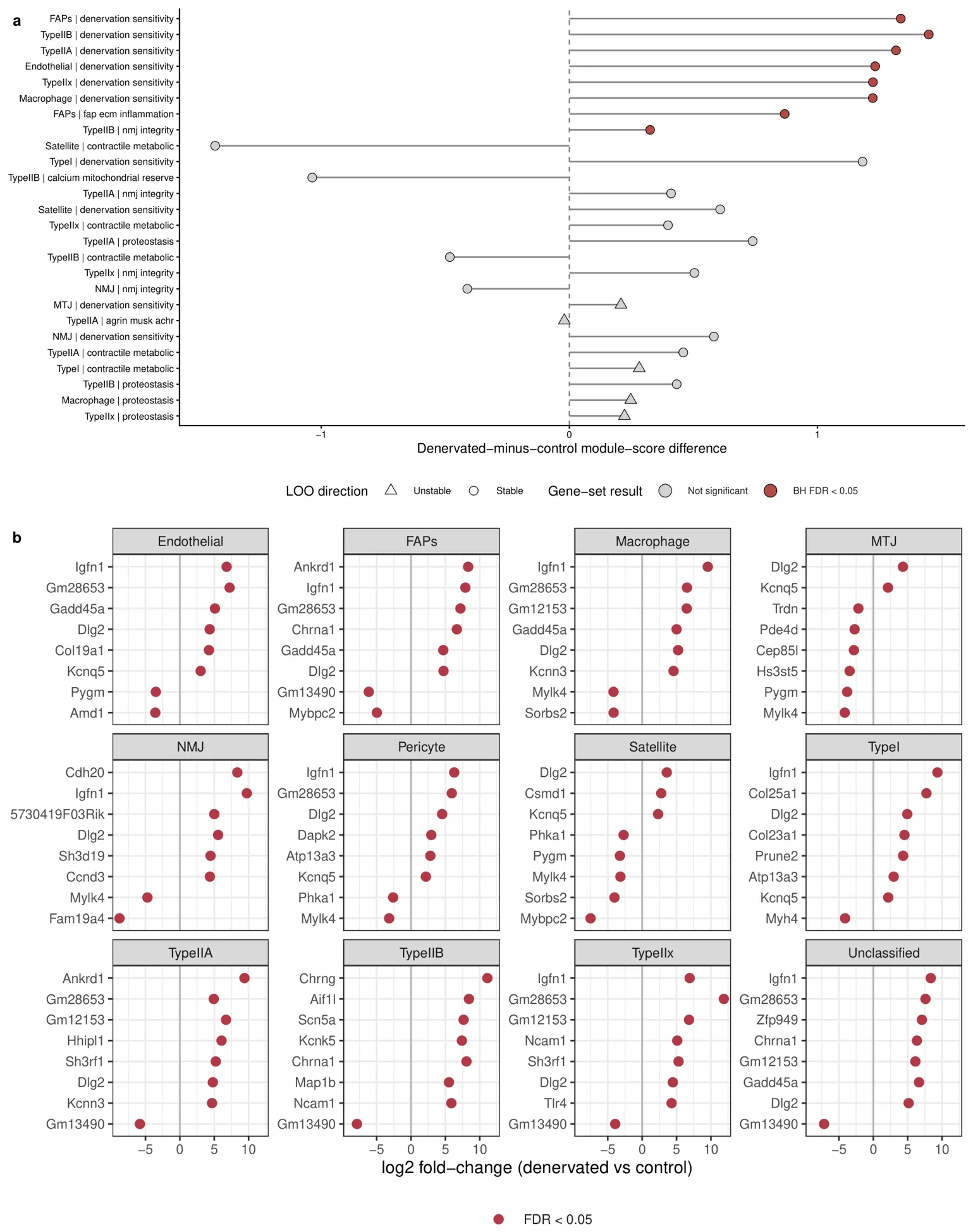
Animal-pseudobulk analysis resolves the denervation response by compartment. (**a**) Direction and FDR of competitive gene-set tests across the 34 analysis-defined applicable compartment–programme combinations. Tests used animal-pseudobulk expression; outlined or labelled entries denote BH FDR below 0.05. (**b**) Leading differential genes from edgeR quasi-likelihood analyses within author-defined compartments. Effect sizes are denervated relative to innervated muscle. No cell-level *p* values contribute to the confirmatory claims.

We first analysed the replicated innervated-versus-denervated endpoint in GSE183802 [36]. Counts were aggregated within each author-defined compartment and animal before differential analysis, preserving six animals as the units of replication. Across 12 analysable cell types, 4311 gene-by-cell-type results reached a Benjamini–Hochberg (BH) false-discovery rate (FDR) below 0.05 (Figure 2; Supplementary Data S1). Type IIB myonuclei accounted for 2448 results, followed by unclassified nuclei (703), FAPs (346), type IIA myonuclei (278) and type IIx myonuclei (144). The NMJ-labelled compartment contained 99 significant genes. Thus, the largest number of detected effects occurred in fast-glycolytic myonuclei, but effects were not confined to the contractile syncytium. Unequal nuclear capture and expression-dependent statistical power affect these counts; they are not a formal test of greater biological vulnerability in type IIB than in other compartments.

To test biological programmes rather than isolated genes, we applied competitive gene-set testing to the animal-pseudobulk profiles. Eight of 34 analysis-defined compartment-applicable tests passed BH FDR below 0.05. Denervation sensitivity increased in FAPs, type IIB, type IIA and type IIx myonuclei, endothelial cells and macrophages (*q* ranging from $2.48\times 10^{-22}$ to $2.67\times 10^{-8}$). FAP extracellular-matrix (ECM)-inflammatory activity was also increased (*q* = 0.00540), and NMJ-integrity genes were enriched in type IIB myonuclei (*q* = 0.0445). All eight significant directions were preserved in every leave-one-sample-out analysis. However, sample-deletion stability did not guarantee gene-deletion stability: all six denervation-sensitivity tests remained FDR-supported after every single-gene deletion, whereas FAP ECM inflammation and type IIB NMJ integrity did not (maximum deletion-round *q* = 0.390 and 0.765, respectively; Supplementary Data S10). The robust core therefore concerns denervation-sensitivity transcription across myonuclear, mesenchymal, vascular and immune compartments. The matrix-inflammatory and junctional panel enrichments are secondary, gene-sensitive observations.

### 2.3. Geriatric Injury Repair Comprises Temporally Distinct Cellular and Molecular Trajectories

**Figure 3:**
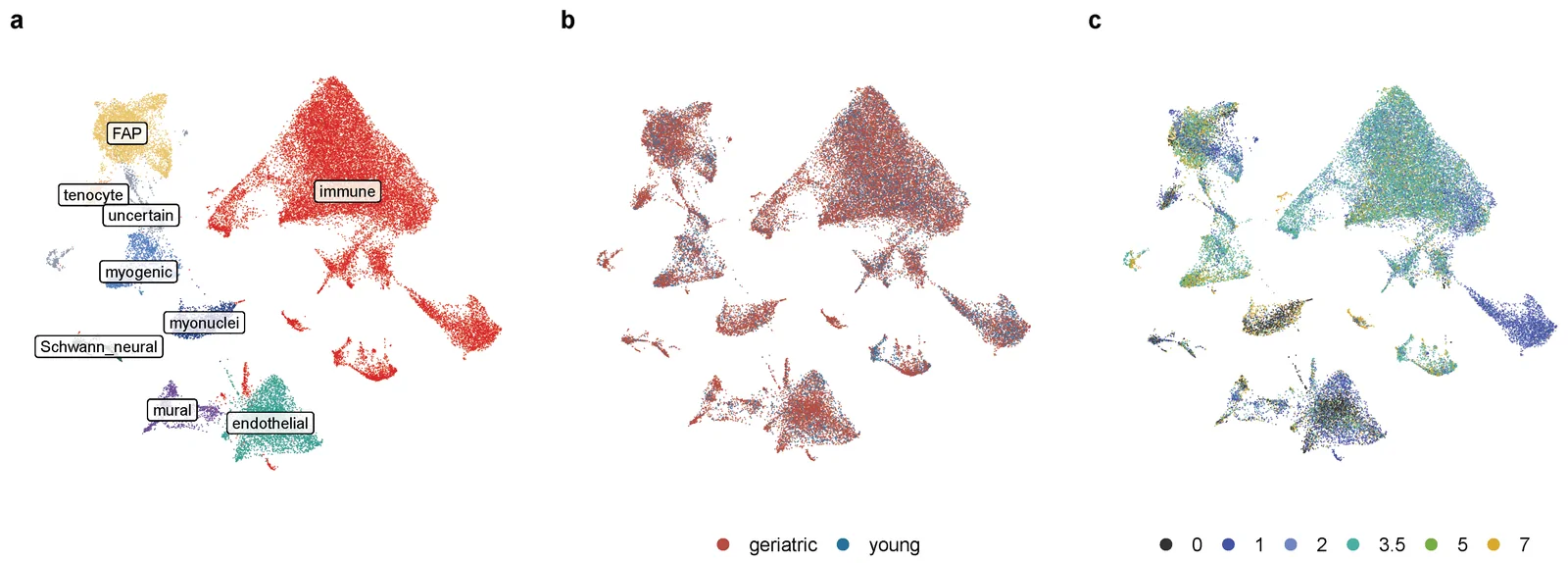
Conservative broad annotation of the local geriatric-regeneration atlas. UMAP representation of 126,977 cells passing the primary fixed-threshold branch, coloured by broad cell class and study variables. Integration was used to facilitate annotation and visualisation; statistical tests were performed after library- or pool-level aggregation. Cells without adequate marker support were retained as uncertain. (**a**) Broad cell compartments. (**b**) Age group. (**c**) Time after notexin injury: the numbers 0, 1, 2, 3.5, 5 and 7 beside the coloured legend dots denote days, with day 0 indicating uninjured baseline. They are not cluster identifiers or cell counts.

We next analysed the locally available GSE232106 regeneration series [37]. It contained 24 geriatric libraries or pools spanning days 0, 1, 2, 3.5, 5 and 7 after notexin injury, together with eight young libraries at days 1 and 3.5. Harmony integration followed by conservative marker-based annotation assigned 126,977 cells across immune, endothelial, FAP, myogenic, myonuclear, mural, Schwann/neural, tenocyte and uncertain classes (Figure 3). The integrated representation separated the principal lineages while preserving contributions from multiple libraries, supporting broad annotation without treating the embedding as an inferential endpoint.

**Figure 4:**
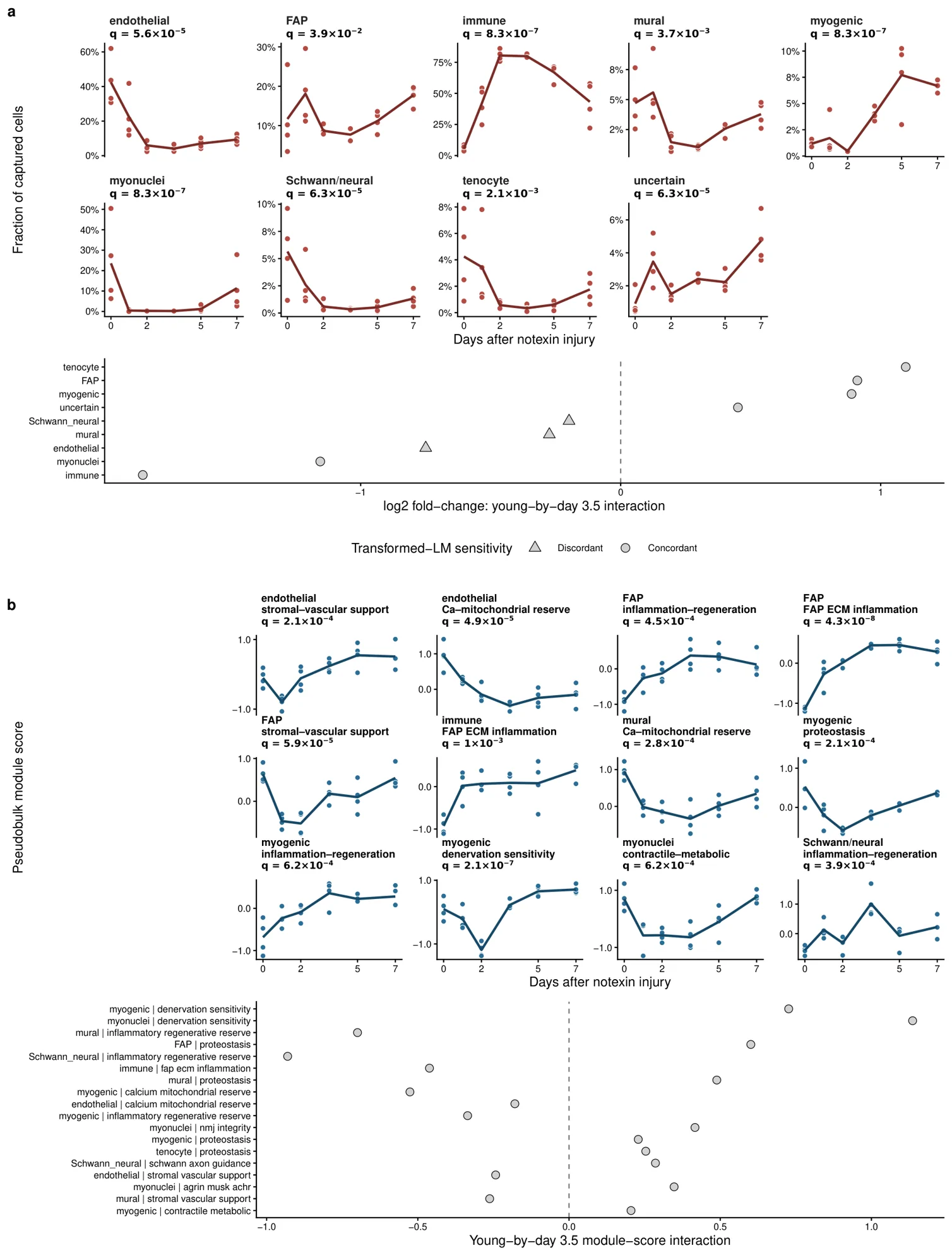
Geriatric muscle regeneration is temporally structured across compartments. (**a**) Library- or pool-level broad-cell composition across the six-point geriatric notexin-injury series, with restricted young observations shown only at available shared days. (**b**) Prespecified pseudobulk programme trajectories within biologically applicable compartments. Omnibus geriatric time tests and restricted age-by-time tests were corrected within their respective families. Points are independent destructive-sampling libraries or pools; coloured lines connect time-point means and do not represent longitudinal tracking of individual cells or animals.

All eight annotated broad compartments varied across the six-point geriatric series after BH correction; the retained uncertain class also varied (Figure 4a). Immune representation expanded after injury, endothelial and myonuclear fractions declined early, and the myogenic fraction increased during subsequent recovery. These shifts occurred at different times and magnitudes, demonstrating that the geriatric response is not a uniform displacement of the tissue along one cellular axis.

Molecular trajectories reinforced this conclusion. Twenty-six of 34 analysis-defined applicable compartment–programme combinations varied across the geriatric time course at BH FDR below 0.05. The strongest effect was FAP ECM-inflammatory activity (*q* = $4.32\times 10^{-8}$), followed by myogenic denervation sensitivity (*q* = $2.15\times 10^{-7}$), endothelial calcium–mitochondrial reserve (*q* = $4.86\times 10^{-5}$) and FAP stromal–vascular support (*q* = $5.89\times 10^{-5}$; Figure 4b; Supplementary Data S2). FAP ECM-inflammatory and myogenic regenerative responses increased after injury, whereas endothelial and energetic programmes showed early troughs and later partial recovery. These findings describe co-occurring but non-identical transcriptional and compositional responses; increased myogenic representation does not by itself establish restored muscle structure or force. Twenty-one of the 26 primary time effects retained FDR support after every gene deletion, and the median-score sensitivity retained 24 of the same 26. FAP ECM inflammation, myogenic denervation sensitivity and endothelial calcium–mitochondrial reserve remained supported throughout deletion rounds, with maximum *q* values of $1.57\times 10^{-7}$, $2.27\times 10^{-6}$ and $4.30\times 10^{-4}$, respectively. Five weaker primary results lost support after at least one deletion: Schwann/neural and mural proteostasis, endothelial inflammatory-regenerative reserve, and myonuclear AGRIN–MUSK–AChR and denervation sensitivity (Supplementary Data S4 and S5).

Additional variance and capture sensitivities retained 23 of the original 26 time effects under Welch tests and 23 after excluding observations with fewer than ten cells (Supplementary Data S12). The FAP ECM-inflammatory, myogenic denervation-sensitivity and endothelial calcium–mitochondrial results remained supported in both. Welch testing lost corrected support for mural proteostasis, myonuclear denervation sensitivity and tenocyte stromal–vascular support; the ten-cell filter lost support for Schwann/neural proteostasis, myonuclear AGRIN–MUSK–AChR and myonuclear denervation sensitivity. Other sensitivity-only significant entries are reported in full but do not become new primary claims. These results support the strongest trajectories while qualifying weaker and sparsely captured compartments.

Restricting programmes to database-confirmed one-to-one pairs retained 25 of the original 26 geriatric time effects and all eight original denervation competitive results (Supplementary Data S14). Mural proteostasis lost corrected time support (q=0.133) after Hspa1a exclusion. This sensitivity improves traceability of the symbol mapping while preserving its uncertainty; it does not establish equivalent programme function across species or supply FDR-supported human validation.

The local source design did not contain a complete young-versus-geriatric factorial trajectory. We restricted age-by-time inference to the shared days 1 and 3.5 and did not borrow information from unobserved young time points. None of nine broad composition interactions or 34 compartment–programme interactions survived BH correction; the smallest module *q* was 0.226. The strongest nominal programme interaction was myogenic denervation sensitivity: estimate +0.726 standardised-score units, pointwise 95% confidence interval 0.243 to 1.210, p=0.00664, *q* = 0.226, with four libraries in each of the four age-by-day groups. Myonuclear combinations instead used three geriatric day-1 libraries because one had no captured myonuclei; their group-specific counts are reported explicitly. Complete estimates, intervals and group sizes accompany all 34 programme interactions and all nine composition comparisons (Supplementary Data S3 and S6). A pointwise interval excluding zero does not override the FDR result. This restricted two-day comparison neither estimates a full young recovery trajectory nor establishes equivalence of young and geriatric responses; it provides no corrected support for an age-dependent change over the observed interval.

### 2.4. Human Ageing and Exploratory Sex Associations Remain Hypothesis-Generating

We then asked whether programme directions identified in mouse perturbations were detectable in two independent human datasets. In E-MTAB-13874 [9], donor-level broad composition was modelled with adjustment for sex and each donor’s nucleus fraction, accompanied by cell-only and nucleus-only sensitivity analyses. None of 13 broad-composition tests passed BH correction (minimum *q* = 0.214). Directional sensitivity differed for some classes by modality, indicating that cells and nuclei should not be treated as exchangeable observations.

**Figure 5:**
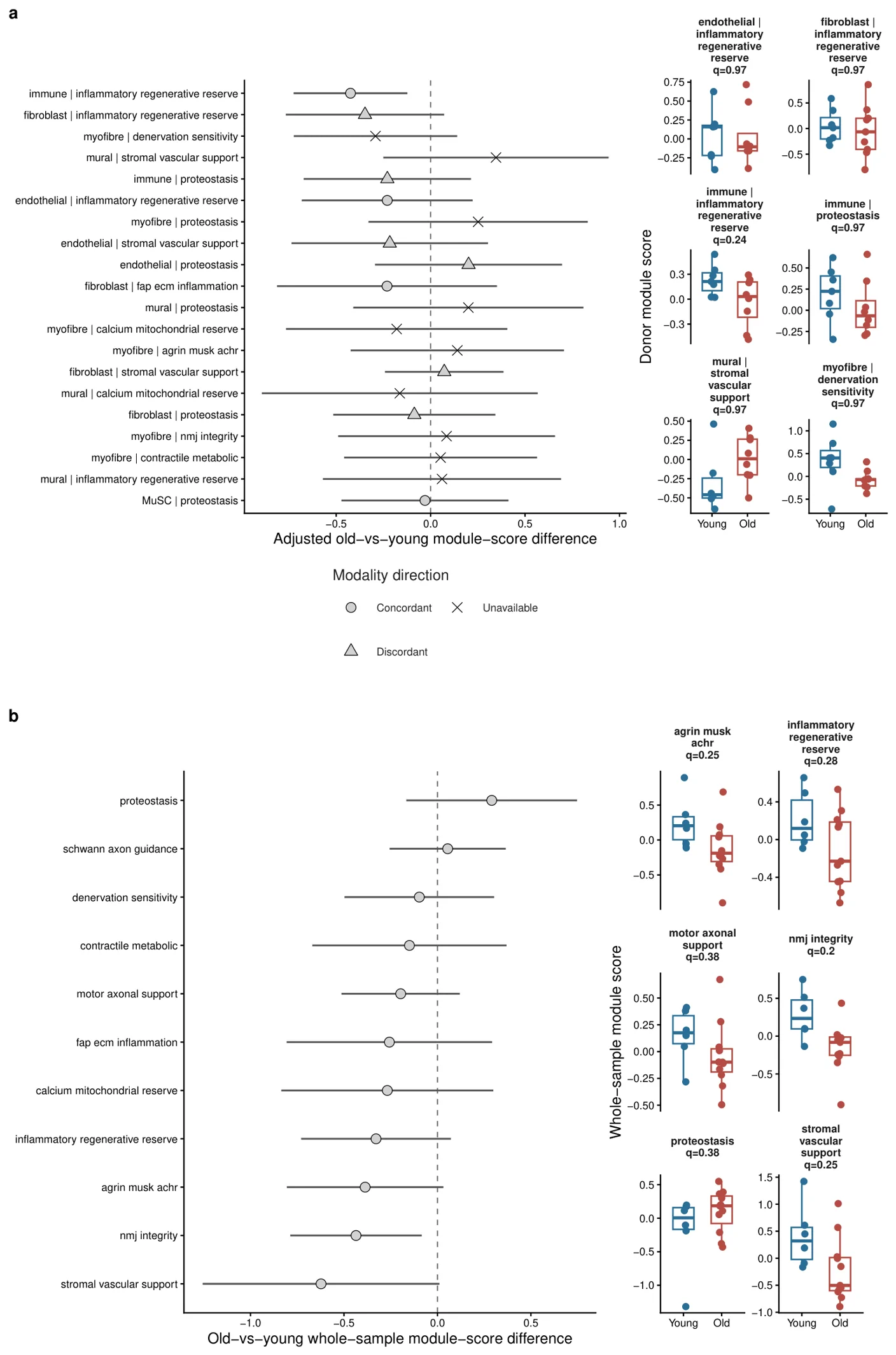
Donor-level hypothesis-generating programme associations in human ageing. (**a**) Adjusted old-versus-young programme estimates in E-MTAB-13874 broad compartments. Primary models account for sex and donor nucleus fraction; sensitivity analyses separate cell and nucleus modalities. (**b**) Old-versus-young whole-sample programme estimates in GSE167186. Points and intervals are donor-level model estimates; nominal *p* values are accompanied by BH-adjusted *q* values. In the box plots, blue denotes young donors and brown denotes old donors. No displayed human programme passed the analysis-defined FDR threshold.

None of 33 analysis-defined compartment–programme tests passed correction in this cohort (minimum *q* = 0.243; Figure 5a). The strongest nominal association was lower inflammatory-regenerative reserve in the immune compartment of older donors (estimate =−0.424, p=0.0101, *q* = 0.243). Rare NMJ and tenocyte compartments did not meet the donor-count threshold for confirmatory testing. In GSE167186 [38], a donor-level whole-sample analysis supplied an independent assay of all 11 programmes. Again, none survived correction. NMJ integrity was nominally lower in older donors (estimate =−0.436, 95% confidence interval −0.787 to −0.084, p=0.0186, *q* = 0.204), with negative directions for stromal–vascular support and AGRIN–MUSK–acetylcholine-receptor organisation (Figure 5b).

These cohorts are relevant because their donors and assays are independent, not because their nominal directions license a replication claim. Selected human effects were biologically compatible with reduced junctional or inflammatory-regenerative capacity, but neither cohort supplied FDR-significant validation. The interpretation is therefore hypothesis-generating: neither the 33 compartment-specific tests nor the eleven whole-sample tests supplied FDR-supported human validation. Nominal directions do not independently confirm mouse findings, and non-significance does not establish absence of an age effect. In the additional E-MTAB-13874 sex analysis, none of the 33 adjusted male-minus-female associations passed BH correction (minimum *q* = 0.134). The nominally strongest effect concerned MuSC inflammatory-regenerative reserve (estimate = −0.588, pointwise 95% confidence interval −0.981 to −0.195, p=0.00720, *q* = 0.134; six female and nine male donors contributing to that compartment). This is an exploratory main-effect estimate, not evidence of a sex-specific ageing or inflammatory response (Supplementary Data S7).

### 2.5. A Preserved Perturbation Null and a Descriptive Exercise Projection Delimit Mechanism

GSE296807 combines nerve-crush sampling with a muscle-directed CRISPR-AAV perturbation targeting CHCHD2 and CHCHD10 [39]. Twenty count matrices were locally available, including 14 replicated PBS or CRISPR-AAV samples at days 0 and 14. Across 11 analysis-defined whole-sample programme interactions, none passed BH correction (minimum nominal p=0.0589; minimum *q* = 0.552). The nominally strongest interaction involved the contractile-metabolic programme, but its uncertainty was inconsistent with a confirmatory effect. We therefore retain the non-significant interaction without selecting a favourable programme post hoc. It is not an equivalence test and does not refute CHCHD2/CHCHD10 biology or the functional observations in the source study. A balanced age-by-time analysis was not attempted because the processed archive omitted six young animals encoded in SRA-only multiplexed libraries.

Finally, we projected the programmes into five public GSE162307 exercise aggregates [40]. At 24 h after the final exercise bout, satellite-cell depletion relative to vehicle was associated descriptively with lower denervation sensitivity (−0.684), AGRIN–MUSK–acetylcholine-receptor organisation (−0.510) and contractile metabolism (−0.487), but higher Schwann/axon-guidance score (+0.549). At 4 h, the ordering differed, including lower inflammatory-regenerative reserve (−0.891) in the depleted aggregate. These opposing directions illustrate molecular asymmetry, but one public aggregate per condition cannot support *p* values, confidence intervals or biological-replicate inference. Accordingly, this analysis remains a projection and is not used to substantiate exercise efficacy or rehabilitation mechanism.

### 2.6. A Cross-Context Evidence Ledger Reveals Programme Specificity

**Figure 6:**
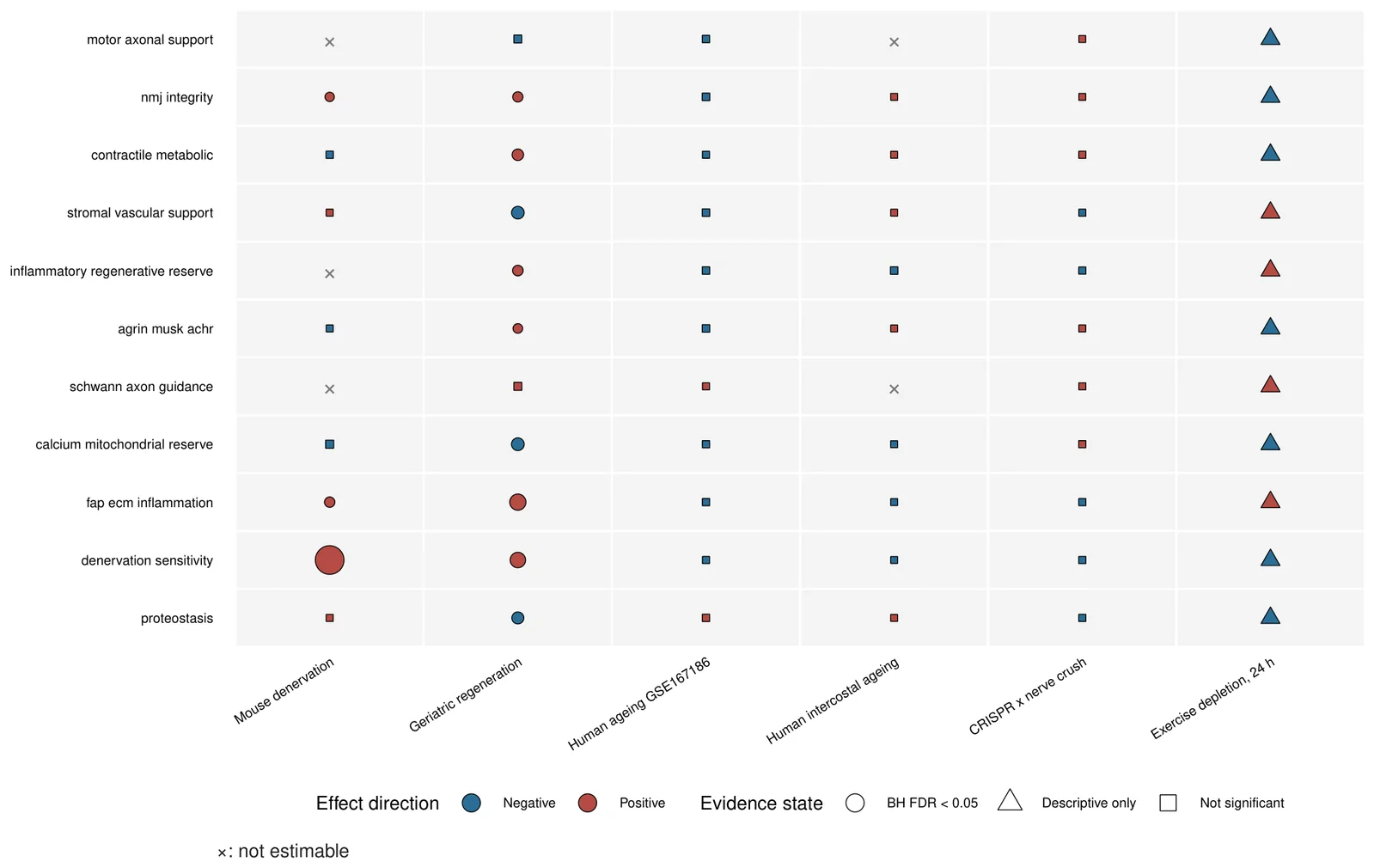
Cross-context evidence map. Signed programme estimates are shown with their cohort-specific evidence class and analysis-defined primary compartment. Symbols distinguish FDR-supported, non-significant, descriptive and non-estimable entries. The display is an evidence ledger, not a pooled meta-analysis: cross-sectional human ageing, mouse denervation, geriatric time, CRISPR-by-injury interaction and aggregate exercise contrasts retain their original estimands and statistical units. For geriatric regeneration, the displayed sign is the descriptive day-7-minus-day-0 mean score difference, whereas the adjusted significance tests any variation across all six days; it is not a corrected test of that endpoint difference. The CRISPR interaction sign is the PBS change minus the CRISPR-AAV change between days 0 and 14.

To compare evidence without meta-analysing biologically incomparable contrasts, we constructed a module-by-context ledger. For every cohort, the display retains the analysis-defined primary compartment, original contrast, statistical unit, signed effect, adjusted significance and non-estimable states (Figure 6). Three mapped denervation results and nine geriatric time-course results passed their cohort-specific BH thresholds. In contrast, none of 11 GSE167186 ageing, nine estimable intercostal-ageing or 11 CRISPR-by-nerve-crush results passed correction; two intercostal combinations were non-estimable and all 11 exercise contrasts remained descriptive.

Directions depended on context. NMJ-integrity activity increased in type IIB nuclei after denervation and across geriatric day 7 relative to day 0, but was lower in older GSE167186 whole-muscle profiles and in the 24 h satellite-cell-depleted exercise aggregate. FAP ECM-inflammatory activity increased after denervation and during geriatric regeneration, yet was directionally lower in both human ageing analyses. These comparisons do not establish conserved reversal because compartment, perturbation and scale differ. Instead, they reveal the central biological pattern: programme identity is insufficient without its cellular location and experimental contrast. The replicated denervation and geriatric time-series designs support compartmental transcriptional remodelling. Their comparison suggests context dependence, but heterogeneous designs and the absence of corrected human or perturbation support do not establish a universal rule about neuromuscular recovery.

### 2.7. Exploratory Communication and Subcellular Evidence Refine the Resource

CellChat predictions were generated separately for all 24 geriatric libraries or pools, preserving biological sampling units rather than constructing one pooled network per time point. The full recurrence ledger contains 2372 day-specific source–target–interaction entries with at least one screened prediction, of which 1281 recur in at least three libraries (including self-directed predictions; Supplementary Table S4). These totals are algorithm-dependent descriptions, not counts of experimentally established interactions. FAP-to-immune CXCL12–CXCR4 was predicted in all four libraries at each injured day. FAP-to-myogenic CXCL12–CXCR4 was predicted in three of four libraries at day 3.5 and four of four at day 5 (Figure S1). Such predictions suggest candidate niche signals for testing; differing capture rates, gene expression and group definitions prevent interpretation as a demonstrated change in signalling strength or causal coordination.

The subcellular extraction showed mixed directions within structural and receptor-related processes. In type IIB nuclei, *Chrng*, *Chrna1* and *Musk* increased (log_2_ fold changes +11.106, +8.065 and +1.556), whereas *Chrne* and *Ache* decreased (−1.715 and −2.888); all passed the original within-cell-type FDR threshold. This pattern is compatible with denervation-associated receptor remodelling, not a uniformly restored adult postsynapse. DGC-associated *Dag1*, *Sgcd* and *Snta1* decreased while *Utrn* and *Dtna* increased. Calcium-handling genes *Ryr1*, *Atp2a1* and *Casq1* decreased. *Atf6* increased, whereas endosomal genes *Rab5a* and *Rab7* changed in opposite directions. These individual RNA effects do not demonstrate DGC disassembly, AChR clustering, mitochondrial respiration, unfolded-protein-response activation or receptor-recycling flux. All selected genes, including non-significant and unavailable results, are retained in Supplementary Data S9; a missing filtered result is not zero expression.

The independent PXD036993 comparison retained 4714 uniquely assigned protein genes, of which 191 overlapped the tested Satellite-compartment RNA output. Of these 191, 120 had the same change direction and 71 differed; no correlation or concordance significance test was assigned. Among the 21 overlapping RNA-FDR-supported genes, 14 agreed in direction and seven did not. For example, *Ncam1* increased and *Sgcd* decreased in both assays, but *Cmya5* and *Fkbp5* differed in direction. This is limited contextual agreement, not validation of the transcriptomic programmes: the protein and RNA measurements differ in cell isolation, anatomical source, time after denervation and replication, and the protein report’s source statistics are not new biological-replicate inference (Supplementary Data S11; Supplementary Table S6).

The second protein comparison retained all 18 selected source proteins, including two neurofilaments without eligible RNA tests. Sixteen had RNA estimates after documented alias resolution: eight directions agreed and eight disagreed (Supplementary Data S16; Figure S3). Ncam1 increased and Mpz decreased in both contexts. By contrast, protein Rab7a increased after chemical sympathectomy whereas its RNA alias Rab7 decreased after sciatic denervation (whole-nuclear log_2_ fold change −1.925, RNA $q=3.94\times 10^{-4}$); Lamp1/2 also lacked a concordant RNA increase. These selected comparisons show why independent protein data can qualify transcriptional interpretation but cannot be presented as uniform replication. Figure S2 provides all seven exploratory subcellular-component RNA panels in type IIB nuclei, with unavailable and non-significant genes retained; Supplementary Table S7 separates transcriptional observations, independent published assay context and the direct measurements still required for each of the five processes requested by the reviewer.

## 3. Discussion

This study identifies two strong, complementary forms of neuromuscular remodelling. Acute denervation produced a large and leave-one-sample-out-stable transcriptional response centred on type IIB myonuclei, with concurrent denervation-sensitivity responses in FAP, macrophage and endothelial compartments. Geriatric injury repair produced a different pattern: all eight annotated broad compartments varied over time, while stromal-inflammatory, myogenic and vascular-energetic programmes followed distinct trajectories. Together, these findings support a model in which neural disruption and tissue regeneration are distributed across specialised cellular compartments. Whether these co-occurring responses are functionally coordinated remains a hypothesis; this analysis did not measure causal coupling between compartments.

The numerous denervation-responsive genes detected in type IIB myonuclei motivate a fibre-type-specific hypothesis and are compatible with spatial specialisation of nuclei within the myofibre syncytium [10,11,41]. However, unequal compartment capture and testing power preclude interpreting differential-gene counts as a direct ranking of fibre vulnerability. The response extended beyond myonuclei. Increased denervation-sensitivity activity in endothelial cells and macrophages and increased ECM-inflammatory activity in FAPs indicate that loss of neural input is registered by the tissue niche, consistent with the established multicellular control of muscle repair [42]. The NMJ-integrity enrichment in type IIB nuclei should not be interpreted as restored synaptic function: transcriptional induction after denervation may represent compensation, remodelling or expansion of a specialised nuclear state. Endplate occupancy requires presynaptic–postsynaptic co-labelling to quantify innervated, partially occupied and vacant endplates; neuromuscular transmission requires electrophysiology, such as evoked responses and repetitive-stimulation measurements; motor-unit activity requires motor-unit recording or number estimation; and force requires direct muscle or in vivo performance testing. None of these endpoints was assessed in the present reanalysis, and functional measurements reported by source investigators were not paired to our pseudobulk estimates as validation outcomes. Larger junctional RNA scores can therefore coexist with impaired innervation or compensatory receptor expression.

The geriatric time series provides a second level of organisation. Early immune expansion and endothelial/myonuclear depletion were followed by increasing myogenic representation, while the most significant molecular trajectories arose in FAP ECM-inflammatory, myogenic denervation-sensitivity and endothelial energetic programmes. FAP activation is therefore not a peripheral accompaniment to regeneration; experimental studies establish these cells as both pro-regenerative niche components and potential sources of pathological fibro-fatty tissue [22,24,43,44]. Yet a beneficial-versus-pathological label cannot be assigned from transcript abundance alone. Transient matrix and inflammatory activation can support repair, whereas persistence can promote fibrosis. The relevant result here is the timing and compartment specificity of the response, not an assumed functional polarity.

**Figure 7:**
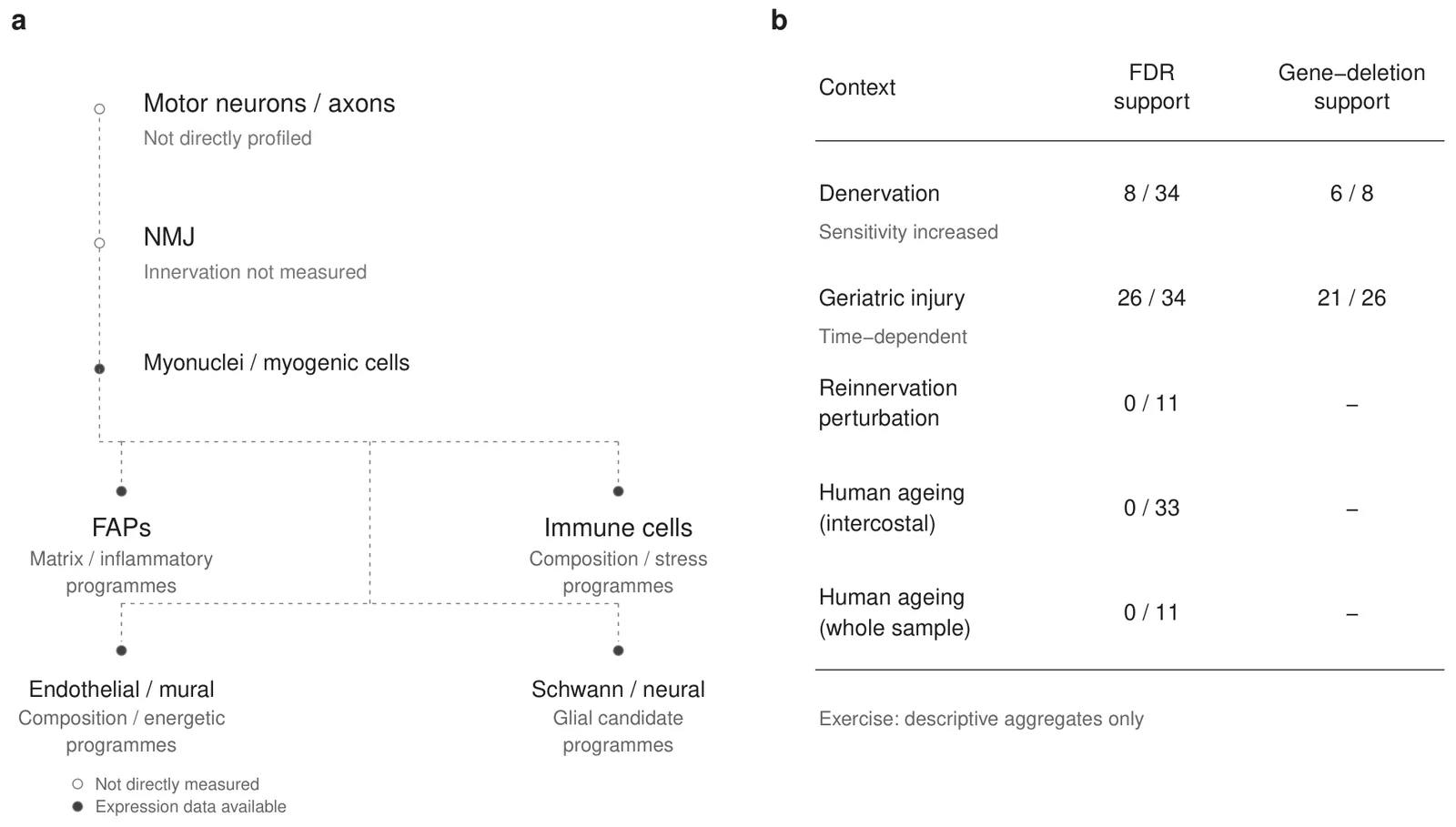
Proposed model of compartment-specific neuromuscular remodelling during denervation and geriatric regeneration. (**a**) Conceptual relationships among neural, postsynaptic, myogenic and niche compartments. Open nodes identify motor neurons/axons that were not directly profiled and the NMJ, whose innervation was not directly measured. Filled nodes identify cell compartments represented in the expression data; these symbols distinguish measurement coverage, not the presence or absence of innervation. Continuous dashed, undirected links indicate conceptual relationships and do not establish signalling or causality. (**b**) Numbers of tests passing the original within-family BH FDR threshold of 0.05. Gene-deletion support counts primary significant results that remain supported after every single-gene deletion: six increased denervation-sensitivity tests and 21 geriatric time effects. The denervation NMJ and FAP-ECM enrichments are gene-sensitive. A dash indicates that this sensitivity was not performed for that context. Reinnervation tests compare PBS with CHCHD2/10 perturbation, not normal recovery or equivalence. Time effects denote variation, not a shared recovery direction; exercise is descriptive. No NMJ occupancy, transmission, motor-unit activity or muscle force was measured.

The proposed compartmental synthesis is shown in Figure 7.

These observations refine the concept of recovery. A shared transcriptional recovery hypothesis would require comparable contrasts, matched cell compartments and a demonstrated relationship between programme changes and functional improvement. The current resource does not satisfy those conditions across all six contexts. Observed directions differed, but the estimands and scales also differed, so discordance alone cannot disprove a conserved mechanism. Junctional, stromal, inflammatory, mitochondrial and contractile programmes could diverge within the same contrast, and the positive mouse findings did not become FDR-significant human ageing associations. A possible systems-level recovery phenotype involving neural, synaptic, myogenic, vascular and stromal states is therefore proposed for future testing (Figure 7), rather than established by the present comparison. In Figure 7, the open motor-neuron/axon and NMJ nodes mark the absence of direct neural profiling and endplate-innervation measurements, respectively; they do not indicate absent innervation in all samples. NMJ-related transcripts were analysed, but their abundance does not establish whether a motor axon occupies an endplate. This formulation is experimentally testable: coordination scores should predict force, motor-unit stability or NMJ occupancy more accurately than individual module scores in a prospectively replicated study.

The non-significant and unavailable results delimit the supported conclusion. The six-point geriatric analysis strongly supports temporal remodelling, whereas the restricted young-versus-geriatric interaction tests address a narrower question and were under the constraint of only two shared time points. Similarly, nominally lower human NMJ and inflammatory-regenerative scores are useful directional observations, but corrected inference does not support cross-species replication. The CRISPR-by-nerve-crush tests also failed to establish a programme interaction in the locally available matrices. Presenting these outcomes alongside their denominators, effect sizes and *q* values prevents a large number of cells from being mistaken for a large number of independent subjects.

Mechanistic interpretation must also distinguish the extracellular cue, the cytoplasmic response and the nuclear transcript. Agrin–LRP4–MuSK signalling organises postsynaptic receptor complexes through protein interactions and phosphorylation, while the DGC provides a membrane-associated scaffold [2,12]. Muscle-specific Yap deletion has experimentally impaired AChR organisation and reinnervation, supporting a role for Yap in NMJ formation and regeneration [45]. In a separate cardiac model, agrin modulated DGC-associated signalling and promoted nuclear Yap localisation [46]; this provides cross-tissue mechanistic context, not evidence that the same agrin–Yap coupling occurred in our skeletal-muscle samples. Hippo kinases regulate the localisation of YAP/TAZ, whose nuclear interaction with TEAD factors can influence myogenic transcription. These are distinct regulatory layers: *Agrn*, *Musk* or *Tead4* RNA does not reveal agrin isoform activity, MuSK phosphorylation, YAP nuclear entry or TEAD occupancy. The increased type IIB *Tead4* transcript in our exploratory extraction (log_2_ fold change +3.247, within-cell-type *q* = $9.67\times 10^{-5}$) motivates a localisation and perturbation test, not a claim that agrin activates YAP in these samples. Similarly, *Atf6* RNA does not measure ATF6 cleavage, and *Xbp1* gene counts do not resolve its spliced active isoform. Receptor-recycling conclusions require surface/internal receptor measurements and trafficking assays, while calcium–mitochondrial interpretation requires calcium handling, respiration and morphology measurements. These distinctions add mechanistic resolution while preventing RNA panels from being equated with subcellular function.

mTOR offers a complementary hypothesis linking protein turnover, metabolism and myogenic state [47]. Muscle-specific Raptor deletion, unlike Rictor deletion in the same model, produced metabolic abnormalities and dystrophic muscle, illustrating that the consequences of disrupting the two complexes depend on the experimental setting [48]. mTORC1, containing Raptor, couples nutrient and growth cues to S6 kinase and 4E-BP1-dependent translational regulation and can restrain ULK1-dependent autophagy. Consequently, transient anabolic signalling during regeneration and persistent suppression of quality-control turnover during ageing need not have the same consequence. mTORC2, containing Rictor, participates in AKT-associated metabolic and cytoskeletal regulation; the two complexes should not be treated as interchangeable [47]. The reviewer-recommended nutrient-signalling studies provide complementary context: amino-acid–Rab1A–mTORC1 signalling can regulate pancreatic PDX1 stability and nuclear localisation [49], and mTORC1-dependent phosphorylation of PDX1 at S61 links nutrient sensing to beta-cell transcription, proliferation and metabolic outcomes [50]. These pancreatic findings illustrate a route from cytoplasmic kinase activity to nuclear regulation, but do not establish the same mechanism in skeletal muscle. The cross-species dietary-restriction review places mTOR alongside insulin/IGF-1, AMPK and sirtuin pathways in the regulation of autophagy, proteostasis, mitochondrial adaptation and longevity, while emphasising context dependence by sex, age and intervention [51]. Its longevity framework should not be equated with a tested muscle-recovery mechanism. The relevant follow-up is to resolve timing and compartment specificity of synthesis, autophagic flux and energetic adaptation rather than infer a uniformly beneficial or detrimental “mTOR score”. In type IIB nuclei, *Mtor*, *Rptor*, *Akt1* and *Tsc2* transcripts increased in the exploratory extraction, but this combination does not establish either complex’s kinase activity: an inhibitor and pathway components can change together without specifying net signalling. The eleven original programmes were not designed as mTORC1/mTORC2 activity assays. No phospho-S6 kinase, phospho-4E-BP1, phospho-AKT, protein-synthesis or autophagic-flux measurements were made. mTOR is therefore a mechanistic framework to test alongside denervation, proteostasis and regeneration, not an additional validated mechanism of this reanalysis.

Independent experiments sharpen the subcellular hypotheses. Lang et al. [52] measured denervation-dependent gastrocnemius protein abundance and turnover, demonstrating that protein stabilisation can contribute to changes that RNA cannot predict. Straka et al. [53] combined proteomics with AChR labelling, immunoblotting and microscopy to document receptor turnover and endo/lysosomal marker redistribution after chemical sympathectomy. Those experiments support the relevance of measuring protein turnover and localisation; they were performed by the source investigators under different conditions and are not new experiments or localisation measurements on our samples. Opposing Rab7-related directions across the compared assays may reflect the different neural perturbations, tissue composition or post-transcriptional regulation; the present data cannot distinguish these explanations.

The analysis has several connected limitations. First, purposive reuse of public studies creates publication and availability bias; differences in muscle source, species, age definition, modality and local completeness remain confounded with biological context (Table 1). Human intercostal muscle is not anatomically matched to lower-limb muscle, and acute mouse injury is not chronic human ageing. Second, destructive sampling does not yield within-animal recovery trajectories. The female-only geriatric injury and male-only denervation experiments cannot identify sex effects, and the exploratory intercostal sex analysis lacks corrected support and does not establish sex-dependent atrophy or inflammation. Third, the restricted GSE232106 interaction covers only two shared days; absent young baseline and late-recovery observations preclude full recovery-kinetics inference. Missing independently processed young matrices in the locally audited GSE296807 snapshot and aggregate-only GSE162307 data further constrain the factorial questions. Fourth, neither human cohort supplied FDR-supported programme validation. Limited donors, capture differences and uncertainty do not justify treating nominal directions as replication or non-significance as equivalence. The GSE167186 age-only whole-sample model additionally leaves sex, frailty and other donor characteristics unadjusted; it does not isolate ageing from potential confounding or changes in cell composition. Fifth, author-curated panels, symbol-case species mapping, overlapping genes and gene-sensitive secondary enrichments constrain specificity. Finally, RNA, CellChat predictions and the descriptive cross-study protein comparison do not measure NMJ occupancy, transmission, motor-unit activity, force, fibrosis or rehabilitation benefit. The six robust denervation-sensitivity results and the strongest geriatric time effects support a transcriptional resource; a causal coordination or recovery mechanism remains unvalidated.

A decisive follow-up would combine repeated electrophysiology, force and motor-unit measurements with terminal single-nucleus and spatial profiling in factorial age, denervation and rehabilitation arms. Perturbations should separately target junctional energetics and stromal inflammation, and analysis plans should prespecify subject-level pseudobulk, interaction contrasts and functional mediation tests. Human validation should prioritise adequately powered donor cohorts with matched biopsy site, cell/nucleus modality and quantitative innervation phenotypes. Such experiments could determine whether compartmental coordination is causal, prognostic or merely correlative.

## 4. Materials and Methods

### 4.1. Study Identification, Acquisition and Provenance

We assembled six public single-cell or single-nucleus datasets: two cross-sectional human ageing cohorts and four mouse studies of denervation, injury-induced regeneration, nerve-crush reinnervation with mitochondrial perturbation and exercise. Because they differed in species, sampling design, perturbation and inferential unit, the studies were analysed as complementary contexts rather than as technical replicates. The resource was purposively assembled to cover these biological contrasts; it was not a systematic search of every eligible muscle atlas. The practical eligibility requirements were public processed expression data, identifiers permitting the stated level of aggregation, interpretable condition or time metadata, and a muscle ageing, innervation, injury or exercise context. These requirements describe the scope of the assembled resource, not a prospectively registered screening protocol. Replication determined the evidence tier, not eligibility: aggregate-only exercise data were retained explicitly as descriptive. Dataset inclusion was not based on obtaining a significant programme result. Nevertheless, publication, archive availability, local-subset selection and unequal anatomical or demographic coverage can bias the resource. It cannot estimate the prevalence of responses across all muscle studies. Table 1 and Supplementary Table S1 compare the designs and their claim boundaries.

GSE167186 provided 100,947 retained profiles from 17 donors for donor-level whole-sample ageing analysis [38]. E-MTAB-13874 provided 183,161 retained cell and nucleus profiles from 17 intercostal-muscle donors for broad-compartment composition and compartment-specific programme analyses [9]. Models for this mixed-modality cohort included sex and donor nucleus fraction as covariates, with cell-only and nucleus-only sensitivity analyses.

GSE183802 contained 26,031 retained nuclei from six animals and provided a replicated innervated-versus-denervated comparison within author-defined compartments [36]. The local GSE232106 regeneration subset comprised 32 libraries or pools: 24 geriatric observations across days 0, 1, 2, 3.5, 5 and 7 after notexin injury and eight young observations at days 1 and 3.5 [37]. It supported broad atlas reconstruction and geriatric composition and programme trajectories; age-by-time inference was limited to the two shared days.

GSE296807 combined nerve-crush sampling with muscle-directed CRISPR-AAV perturbation of CHCHD2 and CHCHD10 [39]. The local resource contained 201,810 profiles across 20 matrices; 14 replicated PBS or CRISPR-AAV samples at days 0 and 14 supported treatment-by-day programme models. Six young animals were available only through three multiplexed SRA gene-expression libraries and three paired cell-multiplexing libraries, precluding a balanced local age-by-time analysis. GSE162307 contributed 15,127 profiles across five exercise-condition aggregates involving vehicle or satellite-cell depletion at 4 and 24 h after the final bout [40]. With one aggregate per public condition, this analysis was descriptive and lacked replicate-based uncertainty estimates.

Count matrices and metadata were stored by accession and audited using filenames, byte sizes, checksums and source sample ledgers. The conservative primary branch contained 654,053 profiles, but inference used donors, animals, libraries, pools or processed condition aggregates. GSE232106 was treated as the locally available subset rather than the complete published atlas, and all availability, exclusion and inferential states were carried into the evidence ledger.

### 4.2. Dataset-Specific Quality Control, Integration and Annotation

Expression matrices were represented as SingleCellExperiment objects to provide a common structure for counts, features, profiles and samples [54]. Quality control remained dataset-specific because preprocessing and count representations differed; supplied data types were preserved, and additional procedures were applied only when their assumptions were compatible.

For integer raw counts with sample identifiers, adaptive thresholds considered library size, detected features and mitochondrial fraction, followed by sample-aware doublet detection. Author-filtered or ambient-corrected non-integer objects were not reprocessed with count-dependent doublet procedures.

For GSE232106, the primary quality-control criteria were a minimum of 200 detected genes per cell, a minimum of 750 unique molecular identifiers (UMIs) per cell, and a mitochondrial-read fraction of at most 25%. It retained 126,977 cells for the main atlas, composition and programme analyses; an adaptive sensitivity branch retained 140,084 cells to assess dependence on the fixed thresholds. Publication-provided DoubletFinder labels were unavailable for the local SoupX matrices and were not reconstructed from integrated coordinates.

GSE232106 cells were library-normalised and integrated with Harmony [55], and a shared-nearest-neighbour graph in the corrected space defined unsupervised clusters. Coherent marker patterns identified immune, endothelial, fibro-adipogenic progenitor (FAP), myogenic, myonuclear, mural, Schwann/neural and tenocyte populations. Clusters with inadequate or incompatible marker support were retained as uncertain rather than forcibly labelled.

UMAP displayed the integrated atlas, broad-lineage separation and library contributions [56], but was not an inferential endpoint. Statistical analyses returned to original library or pool membership and used aggregated composition or expression quantities.

### 4.3. Cell-Composition Analysis

Cell-composition analysis tested whether the representation of a broad cellular class changed across the relevant biological contrast. Cells were counted within each broad class and donor, library or pool, producing one set of compartment counts for each biological sampling unit. Minimum donor-representation and cell-count rules were applied before modelling so that rare compartments with inadequate support were recorded as non-estimable rather than analysed using sparse observations.

For GSE232106, geriatric time effects were tested with edgeR quasi-likelihood negative-binomial models across days 0, 1, 2, 3.5, 5 and 7 after notexin injury [57]. The omnibus time test asked whether the abundance of a compartment differed anywhere across the six-point series and did not require a monotonic trajectory. A separate age-by-time analysis compared the temporal change between young and geriatric samples. This interaction was restricted to days 1 and 3.5, the only time points observed in both age groups, and no values were imputed for unavailable young time points.

The human intercostal analysis used E-MTAB-13874 donor-level compartment counts. Primary models adjusted for sex and for the proportion of each donor’s profiles obtained as nuclei. Cell-only and nucleus-only models were fitted as modality-specific sensitivity analyses, while arcsine-square-root linear models provided a sensitivity analysis based on transformed proportions. BH correction was performed within each analysis-defined composition family so that the number of tested compartments was reflected in the reported *q* values.

### 4.4. Pseudobulk Differential-Expression Analysis

Pseudobulk analysis was used to test transcriptional change within a compartment while preserving the biological replicate. Raw gene counts were summed across all eligible cells or nuclei belonging to the same replicate and compartment. Each pseudobulk profile therefore represented a donor, animal, library or pool rather than an individual captured cell. Genes with inadequate expression across replicate pseudobulks were removed before normalisation and dispersion estimation, limiting tests based on isolated or extremely sparse counts.

Differential expression was modelled with edgeR quasi-likelihood methods, with the design matrix defined by the experimental structure of the source dataset [57]. The principal gene-level analysis used GSE183802. Counts were aggregated by animal within the author-defined compartments, and denervated tissue was contrasted with innervated tissue while retaining the animal as the unit of inference. Multiple-testing correction for the complete differential-expression output was performed within cell type, consistent with the cell-type-specific results supplied in Supplementary Data S1. Cell-level differential-expression *p* values were not used for confirmatory claims because the captured cells or nuclei were subsamples of the same biological replicate.

### 4.5. Neuromuscular Programme Definition, Scoring and Statistical Models

We defined eleven complementary gene programmes: motor-axonal support, NMJ integrity, contractile metabolism, stromal–vascular support, inflammatory-regenerative reserve, AGRIN–MUSK–acetylcholine-receptor organisation, Schwann/axon guidance, calcium–mitochondrial reserve, FAP ECM inflammation, denervation sensitivity and proteostasis. These remained separate biological dimensions rather than a single recovery score.

The exact author-curated gene panels are provided in Supplementary Data S13, including human and mouse symbols, panel sizes and existing references that support the biological context. These references do not validate the exact panel as a biomarker or prove that all included genes have the same functional polarity. The eleven panels contain 8–10 genes each and overlap; for example, the eight AGRIN–MUSK–acetylcholine-receptor genes are nested within the ten-gene NMJ-integrity panel. Programme names such as “integrity” and “reserve” are shorthand for candidate transcriptional panels, not direct measures of synaptic integrity or physiological reserve. Mixed panels can contain activation, differentiation and inflammatory genes, so a higher average is not automatically beneficial [58,59].

The analysis scripts and applicability configurations define the original testing families: 34 compartment–programme combinations in GSE232106 and 33 in E-MTAB-13874. Both complete whitelists and their compartment-specific rationales are provided in Supplementary Table S2. Rules assign postsynaptic panels to myonuclei, glial panels to Schwann/neural populations, and matrix, inflammatory or energetic panels to their plausible recipient compartments; they are hypotheses about applicability rather than proven expression specificity. The denervation whitelist is separately defined at the author-labelled cell-type level, with 34 estimable combinations, and is not identical to the regeneration whitelist. The full Cartesian product remains exploratory. All original gene lists, whitelists and primary result families were retained unchanged during revision; sensitivity tests were labelled separately and did not replace the primary results. The available scripts document fixed definitions for the reported workflow but are not evidence of external preregistration, independent validation or blinding before every original exploratory analysis. We therefore use “analysis-defined” rather than “prespecified” for these original candidate families. Revision-stage freezing prevents adapting the lists to the additional analyses but cannot retrospectively establish outcome-independent initial selection; programme-level findings remain exploratory biological hypotheses even when they pass the reported within-family FDR threshold.

For count-based cohorts, programme scores were the arithmetic mean of gene-wise standardised pseudobulk log-counts per million, after library normalisation with a prior count of 2. Genes absent from the expression matrix were excluded; at least three panel genes were required. Zero-variance or non-finite standardised values were set to zero, as in the original scoring script. This gives variable genes equal scale rather than weighting by constitutive abundance, but does not remove low-expression noise, panel overlap or mixed biological polarity. In E-MTAB-13874, the supplied processed expression assay was averaged within donor, compartment and modality; its genes were standardised across eligible donor–modality groups, and modality scores were combined by cell-number-weighted means before donor modelling. This differs from raw-count pseudobulk and is an additional comparability limitation. Supplementary Table S3 reports gene availability and variability for each primary geriatric combination.

The original species conversion used symbol case: uppercase human symbols and initial-uppercase/rest-lowercase mouse symbols. It did not perform a database-verified one-to-one orthologue mapping. Gene availability, paralogy, capture modality, anatomical source and compartment annotation can therefore change the meaning and sensitivity of a panel across cohorts. Cohort-specific standardisation centres and scales each gene within that cohort; equal score differences are not equal molecular effects across technologies or species. Whole-sample scores can additionally reflect cell composition. Cross-study direction comparisons are qualitative assessments of distinct estimands, not biological replication or a calibrated cross-species effect scale.

GSE183802 programme inference used camera competitive tests on animal-pseudobulk expression, retaining the animal as the replicate and specifying an inter-gene correlation of 0.01 [60]. BH correction covered the 34 applicable tests. Directional robustness was assessed by omitting each animal in turn and recalculating the denervated-minus-innervated score difference; results were stable only when every contrast retained the full-data sign.

For GSE232106, programme scores were calculated by broad compartment and library or pool. Omnibus models tested variation across the six geriatric time points, whereas restricted models used days 1 and 3.5 to test age-by-time interactions. These questions formed separate inferential families. The local design contains four libraries or pools per observed age-by-day group: geriatric females aged 26 months at all six days, and young females aged 4.7 months at days 1 and 3.5. Geriatric pools are the sequencing units and are not expanded into additional mouse replicates. Programme models include only libraries with non-zero compartment counts; thus, compartment-specific sample numbers can be below four. Unlike the gene-level differential-expression filter, these score models do not impose a ten-cell minimum, so sparse compartments have noisier estimates and weaker interpretability. The restricted interaction is $\left(S_{\mathrm{young},3.5}-S_{\mathrm{young},1}\right)-\left(S_{\mathrm{geriatric},3.5}-S_{\mathrm{geriatric},1}\right)$. Ordinary linear models provide the estimate, standard error and pointwise 95% Student-*t* confidence interval, with residual degrees of freedom and all four group sizes in Supplementary Data S3. These intervals are not multiplicity-adjusted; BH correction applies to the 34 interaction tests. Supplementary Data S6 retains original edgeR composition log-fold changes and adjusted values and separately gives pointwise intervals from the arcsine-square-root proportion sensitivity model. Those intervals are not edgeR log-fold-change intervals.

Human ageing associations were tested independently at donor level. E-MTAB-13874 used compartment-specific scores and covariates matched to its mixed cell/nucleus design; GSE167186 used whole-sample scores for all eleven programmes. The cohorts were not pooled. The GSE167186 model included age class alone: sex, frailty and other donor characteristics were not adjusted for, and whole-sample composition can contribute to the estimate. It therefore cannot isolate an age effect independent of these potential confounders.

GSE296807 models tested whether programme changes between days 0 and 14 differed between the replicated PBS and CRISPR-AAV conditions. A balanced age interaction was not fitted because the required young-animal matrices were unavailable as independent processed samples. The reported signed coefficient is $\left(S_{\mathrm{PBS},14}-S_{\mathrm{PBS},0}\right)-\left(S_{\mathrm{CRISPR},14}-S_{\mathrm{CRISPR},0}\right)$, following the original model’s CRISPR reference level.

For GSE162307, programme results were arithmetic differences between the five condition-level aggregates, including satellite-cell-depleted versus vehicle comparisons at matched post-exercise times. Because replicate-level variation was unavailable, standard errors, confidence intervals and inferential *p* values were not assigned.

### 4.6. Sensitivity, Communication and Protein Comparisons

To examine gene dependence, we repeated the 34 geriatric tests after omitting each panel gene and separately replaced the mean gene-standardised score with its median. Single-gene deletions were organised into ten deterministic rounds, each containing the complete 34-test family; panels with fewer than ten available genes repeated their final deletion in later rounds. BH correction was applied within each complete round, and the minimum and maximum adjusted values over all deletions were retained. The procedure is a post-review sensitivity analysis, not an exhaustive evaluation of every possible simultaneous deletion across panels. The same procedure was applied to denervation camera tests, using the original animal pseudobulks, filtered gene universe, design and inter-gene correlation. Supplementary Data S4, S5 and S10 preserve both retained and lost support.

To examine cross-species mapping directly, we queried Ensembl REST for each of the 78 unique human panel symbols, resolved the canonical human gene ID to exclude alias hits and retrieved mouse orthologues and target gene symbols (3 October 2026; cached responses in the analysis record). Of 105 programme–gene entries, 102 corresponding to 75 unique symbols matched a one-to-one mouse orthologue. HSPA1A mapped many-to-many to Hspa1a/Hspa1b; NEFM and MYH2 returned no mouse homology record in this API snapshot, which is not evidence that an orthologue is biologically absent. A conservative sensitivity retained only the confirmed one-to-one pairs, excluded these three original mouse symbols, and repeated all 34 geriatric time tests and 34 denervation competitive tests, with separate BH families. Original programmes and primary outputs were not replaced (Supplementary Data S13 and S14).

Additional post-review model diagnostics assessed the geriatric score models without redefining the original family. Welch one-way tests allowed unequal variances among the six day groups. A separate sensitivity excluded compartment–library observations containing fewer than ten cells and refitted the original ordinary time model; all 34 combinations retained at least two observations per day. Scores were taken from the original standardised matrix rather than rescaled after exclusion. BH correction was applied separately across all 34 tests for each sensitivity (Supplementary Data S12). These analyses assess model/capture sensitivity and do not replace primary results or establish new confirmatory discoveries.

CellChat version 2.2.0.9001 [61] was used for an exploratory analysis of predicted secreted ligand–receptor communication in the 24 geriatric GSE232106 libraries or pools. Each library was analysed separately, retaining FAP, immune, endothelial and myogenic broad groups with at least 20 cells. Up to 400 cells per group were sampled reproducibly, full-transcriptome counts were normalised to 10,000 per cell and log-transformed, and the bundled mouse “Secreted Signaling” database was used. Inference used overexpressed genes/interactions, the trimean, unprojected expression, no population-size weighting, no spatial-distance weighting and 100 cell-label permutations. Library-specific predictions passing the software’s nominal permutation threshold of p<0.05 were summarised by recurrence among eligible libraries at each day. There were four libraries per day, but eligibility for a source–target pair varied from two to four because each retained group required at least 20 cells; this denominator is reported for every prediction. These cell-label permutation values are computational screening quantities, not biological-replicate tests of communication or corrected tests of between-day changes. Absence of a prediction means no supported prediction under these settings, not proven absence of signalling. We report all predictions, recurrence denominators and sampled group sizes in Supplementary Data S8 and Supplementary Tables S4 and S5; Figure S1 displays an explicitly selected CXCL12–CXCR4 example. Motor neurons, axonal terminals and synaptic protein localisation were not measured, and broad groups conceal signalling heterogeneity.

An independent public protein comparison used the PRIDE project PXD036993 [62] and its young-denervation-versus-sham protein-candidate report. The source profiles FACS-isolated hindlimb muscle stem cells 21 days after denervation, whereas the closest RNA compartment here is the GSE183802 author-labelled Satellite group in gastrocnemius at 14 days. Only unique single-gene protein assignments were joined to tested RNA genes; ambiguous protein groups and duplicate gene assignments were excluded. Protein log-ratios and source-reported statistics were retained, without refitting tests or treating the four sham and five denervated technical runs as independent animals. The comparison is descriptive and compartment/time-mismatched; it cannot validate type IIB myonuclei, the NMJ, or geriatric regeneration. Source URLs, downloaded-file checksums, exclusions and complete matched results are provided with Supplementary Data S11 and Supplementary Table S6.

For a second protein context focused on receptor trafficking, all 18 entries of the published Table 2 in Straka et al. [53] were transcribed, and UniProt accessions, mouse gene symbols and aliases were verified. The source compares two-week chemical sympathectomy with PBS in whole tibialis anterior, with four muscle specimens per group, and reports nominal protein *p* values. Its table is an author-selected subset of the 2215-protein study, not an unbiased validation set; the protein-identification FDR is distinct from differential-abundance multiplicity control. We added a whole-retained-nucleus GSE183802 pseudobulk aggregated by animal across compartments, using the original QC branch and edgeR quasi-likelihood workflow (three control and three sciatic-denervated animals). Gene filtering, TMM normalisation, robust dispersion estimation and BH correction over all 12,890 tested genes were applied before joining the 18 proteins. Unique source-supported aliases were resolved explicitly, including Rab7a to the RNA label Rab7. Unavailable RNA tests, source nominal statistics and opposing directions were retained. Different nerves, muscles and RNA/protein sampling prevent a matched validation inference (Supplementary Data S15 and S16; Figure S3).

Sex-dependent muscle homeostasis, atrophy and inflammatory responses provide an important biological motivation for reporting sex coverage and testing eligible associations [63]. Sex was a covariate in the primary E-MTAB-13874 ageing model, not a primary sex-specific endpoint. We additionally extracted exploratory male-minus-female main effects adjusted for age class and donor nucleus fraction across its 33 applicable programme tests, with pointwise 95% intervals and BH correction in Supplementary Data S7. These effects do not estimate sex-by-age or sex-by-atrophy interactions. GSE232106 is female-only and GSE183802 is male-only; neither identifies sex differences. Other locally simplified design ledgers do not provide a complete sex-balanced factorial design.

To deepen interpretation without redefining the original primary programmes, we extracted the original GSE183802 gene-level results for seven post-review panels: DGC membrane scaffold, AChR assembly/clustering, calcium–mitochondrial genes, ER stress/unfolded-protein response, receptor/endosomal traffic, Hippo/YAP-related genes and mTOR-complex regulators. Supplementary Data S9 includes every listed gene and compartment, including genes absent from the filtered output, with original within-cell-type FDR values. Panel membership is an exploratory biological annotation, not a new pathway-activation test or evidence of subcellular protein localisation.

### 4.7. Cross-Context Evidence Synthesis and Statistical Reporting

The cohort-specific analyses estimated different quantities: a denervated-versus-innervated effect, a human ageing association, a geriatric omnibus time effect, an age-by-time or treatment-by-day interaction and an aggregate exercise difference are not estimates of the same statistical parameter. We therefore synthesised the results in an evidence ledger rather than pooling them in a meta-analysis. The evidence map used one analysis-defined primary compartment for every programme and cohort using the fixed mapping supplied with the analysis scripts rather than selecting a compartment anew for the revision. This documents the display rule but does not establish that the original mapping was chosen blind to the exploratory results.

Each ledger entry retained the source accession and contrast, the biological unit supporting the analysis, the selected compartment, the signed estimate, nominal *p* value, adjusted *q* value and evidence status. Inferential results passing the relevant BH threshold were classified as FDR-supported; estimable tests not passing correction were classified as non-significant. Aggregate-only comparisons were classified as descriptive, and combinations lacking the necessary samples, donor coverage or factorial cells were marked as non-estimable. This structure allowed positive, null and unavailable evidence to remain visible without treating their numerical outputs as directly exchangeable.

All confirmatory families used the BH procedure to control the false-discovery rate [64]. Correction was applied within the analysis-defined family belonging to each analysis. Results with *q* ≥0.05 were not described as statistically significant even when the nominal *p* value was below 0.05, and exploratory results were not promoted into the confirmatory family after inspection.

Missing libraries, inadequate donor representation and absent factorial cells were recorded as unavailable tests rather than replaced by imputed observations. Cells and nuclei were never treated as independent subjects. Each primary claim was accompanied by the effect estimate or direction, the biological replicate supporting the comparison and the family over which multiplicity correction was performed. This reporting policy prevented the large number of captured profiles from being interpreted as an equally large number of independent experimental units.

### 4.8. Software, Visualisation and Reproducibility

All data processing, statistical analysis and scientific figure generation were performed in R (version 4.4.2). Core packages included SingleCellExperiment, edgeR, Seurat [65], Harmony, Matrix, data.table, ggplot2, patchwork, svglite and ragg.

Tabular source data accompanied the displays so that plotted quantities could be traced to their numerical outputs. An automated submission-artifact validator checked cohort profile totals, statistical-unit counts, principal *q* values, preservation of null and non-estimable results, figure availability and consistency of the cross-context evidence ledger. All 20 original validation checks passed before initial assembly of the manuscript and supplementary outputs. Reviewer-requested analyses were subsequently checked against the unchanged primary statistics and are documented separately in the revision analysis records.

## 5. Conclusions

The replicated analyses support compartment-specific denervation-sensitivity transcription and temporally structured composition and programme changes in geriatric injured muscle. Gene-deletion sensitivity identifies a robust core while qualifying weaker junctional and stromal enrichments. The human ageing, restricted age-by-time and reinnervation-perturbation tests do not supply FDR-supported validation; exercise remains descriptive. These results establish an auditable comparative resource, not NMJ functional recovery, conserved cross-species signalling or a causal requirement for compartmental coordination. The proposed model is a testable hypothesis whose evaluation requires matched biological replication, protein and localisation measurements, and direct neuromuscular functional endpoints.

## Figures and Tables

**Table 1:** Dataset designs and limits of cross-context comparison. Full metadata, availability and provenance are provided in Supplementary Table S1. Ages and sample numbers describe the analysed design or explicitly identified source metadata; cellular coverage is not biological replication.

Accession	Species; Muscle; Modality	Age/Time Scope	Statistical Units	Interpretation Limit
GSE167186	Human; lower limb; nuclei	6 young, 11 old donors	17 donors	Whole-sample, cross-sectional
E-MTAB-13874	Human; intercostal; cells and nuclei	8 young, 9 old donors	17 donors	Site and modality differ; adjusted age associations
GSE183802	Mouse; gastrocnemius; nuclei	Adult male; 14-day denervation	3 control, 3 denervated animals	Replicated endpoint; no recovery trajectory
GSE232106	Mouse; tibialis anterior; cells	Female; 26-month geriatric days 0–7; 4.7-month young days 1/3.5	24 geriatric pools and 8 young libraries	Restricted two-day age interaction; no full young trajectory
GSE296807	Mouse; tibialis anterior; nuclei	Local PBS/CRISPR at days 0/14	14 factorial samples within 20 matrices	Treatment interaction; local young design incomplete
GSE162307	Mouse; exercise soleus/reference aggregates; nuclei	Vehicle/depletion at 4/24 h	5 condition aggregates	Descriptive; no replicate uncertainty

## Data Availability

The public datasets analysed in this study are available from the Gene Expression Omnibus (GEO) under accession numbers GSE167186, GSE183802, GSE232106, GSE296807, and GSE162307, and from the EMBL-EBI BioStudies/ArrayExpress database under accession number E-MTAB-13874. Processed result tables supporting the analyses are provided as Supplementary Materials. The post-review descriptive protein comparisons use the public PRIDE/ProteomeXchange projects PXD036993 and PXD021601, including the published Straka et al. [53] (Table 2 therein); the original source download locations and checksums are supplied in Supplementary Table S6. No new raw sequencing data were generated in this study. The custom analysis scripts are not currently deposited in a public repository but can be made available by the corresponding author upon reasonable request.

## Supplementary Materials

The following supporting information can be downloaded at https://www.mdpi.com/article/10.3390/ijms27209025/s1.
