# Supplementary material for: Compartment-Specific Transcriptional Remodelling Defines Denervation and Geriatric Muscle Regeneration
Source: Int J Mol Sci. 2026 Oct 12;27(20):9025. doi: 10.3390/ijms27209025 (PMC13705149; doi:10.3390/ijms27209025)
Supplement: Supplementary file 1 [file ijms-27-09025-s001.zip › Supplementary.pdf]

Supplementary Materials

Compartment-Specific Transcriptional Remodelling Defines Denervation and Geriatric Muscle Regeneration

Wanting Tong, Qi Yu, Renyu Yang, Chunxue Shao,
Guang Yang, and Ziheng Wang

Contents and data-file notes

This document presents the cohort design and evidence ledger as Supplementary Table S1 (displayed in parts S1a–S1b) and the complete GSE232106 geriatric programme test results as Supplementary Data S2, organized into geriatric time-course and restricted age-by-time sections. The accompanying tab-delimited files provide the machine-readable source tables:

- `Supplementary_Table_S1_study_evidence_ledger.tsv`: six-cohort provenance, retained profile counts, inferential units, evidence grades, analysis domains, and limitations.
- `Supplementary_Data_S1_denervation_differential_expression.tsv.gz`: 34,788 gene-by-cell-type rows from the GSE183802 denervated-versus-innervated edgeR analysis. The `FDR` column is adjusted within cell type; 4,311 rows have FDR below 0.05.
- `Supplementary_Data_S2_geriatric_programme_tests.tsv`: 88 compartment–programme rows containing geriatric time-course and restricted age-by-time results. The 34 biologically applicable tests are identified in the source file; applicable-family adjusted values are retained in the columns ending in `FDR_applicable`.

All source files are tab-delimited. Blank values denote non-applicable or non-estimable results and are not zero effects. The compressed differential-expression file is intentionally supplied as data rather than printed in full in this PDF.

Table S1a: Cohort identity, retained profiles, inferential units, and evidence grade.

Dataset ID	Accession	Study label	Species	Retained profiles	Units (n)	Unit type	Evidence grade
human_ageing_sarcopenia	GSE167186	Human ageing snRNA-seq	Human	100947	17	donors	Cross-sectional
human_intercostal_ageing	E-MTAB-13874	Human intercostal atlas	Human	183161	17	donors	Cross-sectional
mouse_denervation	GSE183802	Mouse denervation	Mouse	26031	6	animals	Replicated endpoint
mouse_lifespan_regeneration	GSE232106	Geriatric regeneration	Mouse	126977	32	libraries/pools	Population time series
mouse_nerve_crush_reinnervation	GSE296807	Nerve-crush reinnervation	Mouse	201810	20	local libraries	Incomplete time series
mouse_exercise_myonuclei	GSE162307	Exercise myonuclei	Mouse	15127	5	condition aggregates	Descriptive aggregate

Table S1b: Analysis domains represented in each dataset (Yes/No) and the primary limitation recorded in the evidence ledger.

Accession	Ageing	Denervation	Injury/recovery	Reinnervation	Exercise	Perturbation	Primary limitation
GSE167186	Yes	No	No	No	No	No	Cross-sectional; simplified metadata supports young versus old only
E-MTAB-13874	Yes	No	No	No	No	No	Mixed cells and nuclei; donor models adjust for modality
GSE183802	No	Yes	No	No	No	No	Endpoint contrast does not identify recovery dynamics
GSE232106	Yes	No	Yes	No	No	No	Six-time series is geriatric only; young overlaps at days 1 and 3.5
GSE296807	Yes	Yes	No	Yes	No	Yes	Six young animals are absent from the GEO matrix archive
GSE162307	No	No	No	No	Yes	Yes	One public aggregate per condition precludes biological-replicate inference

Supplementary Data S2. GSE232106 geriatric programme tests

The following tables reproduce all 88 rows in the source test matrix. The geriatric time-course analysis reports the time-course test and its adjusted values; the restricted age-by-time analysis reports the interaction estimate and tests. Numerical values are rounded for display; the source TSV retains full precision. The unqualified adjusted values cover the reported matrix, whereas the `FDR applicable` values correspond to the prespecified biologically applicable family. Applicability and rationale follow the source file; blank fields are shown as em dashes.

Geriatric time-course results

Compartment	Programme	Genes	Time P	Time FDR	Applicable (Yes/No)	Applicable time FDR	Rationale
FAP	agrin__musk__achr	8	2.25×10^{-4}	9.84×10^{-4}	No	—	—
FAP	calcium__mitochondrial__reserve	10	0.124	0.145	No	—	—
FAP	contractile__metabolic	10	6.41×10^{-7}	1.41×10^{-5}	No	—	—
FAP	denervation__sensitivity	9	1.04×10^{-7}	3.06×10^{-6}	No	—	—
FAP	fap__ecm__inflammation	10	1.27×10^{-9}	1.12×10^{-7}	Yes	4.32×10^{-8}	core stromal-ECM programme
FAP	inflammatory__regenerative__reserve	10	1.19×10^{-4}	6.14×10^{-4}	Yes	4.48×10^{-4}	injury-responsive stromal signalling
FAP	motor__axonal__support	10	0.00318	0.00706	No	—	—
FAP	nmj__integrity	10	7.11×10^{-5}	4.17×10^{-4}	No	—	—
FAP	proteostasis	8	0.00245	0.00582	Yes	0.00488	general stress-control programme
FAP	schwann__axon__guidance	10	0.00467	0.00934	No	—	—
FAP	stromal__vascular__support	10	6.93×10^{-6}	7.62×10^{-5}	Yes	5.89×10^{-5}	stromal and perivascular support genes
Schwann__neural	agrin__musk__achr	8	0.00679	0.013	No	—	—
Schwann__neural	calcium__mitochondrial__reserve	10	0.00761	0.014	No	—	—
Schwann__neural	contractile__metabolic	10	0.00222	0.00542	No	—	—
Schwann__neural	denervation__sensitivity	9	0.0287	0.0402	No	—	—
Schwann__neural	fap__ecm__inflammation	10	0.394	0.418	No	—	—
Schwann__neural	inflammatory__regenerative__reserve	10	9.22×10^{-5}	5.07×10^{-4}	Yes	3.92×10^{-4}	injury-responsive glial signalling
Schwann__neural	motor__axonal__support	10	0.0881	0.109	Yes	0.0999	glial-neural support programme
Schwann__neural	nmj__integrity	10	0.0185	0.0275	No	—	—
Schwann__neural	proteostasis	8	0.0168	0.0269	Yes	0.0248	general stress-control programme
Schwann__neural	schwann__axon__guidance	10	0.0704	0.0912	Yes	0.0855	Schwann and axon-guidance programme
Schwann__neural	stromal__vascular__support	10	0.00321	0.00706	No	—	—
endothelial	agrin__musk__achr	8	2.86×10^{-4}	0.00109	No	—	—
endothelial	calcium__mitochondrial__reserve	10	4.29×10^{-6}	5.39×10^{-5}	Yes	4.86×10^{-5}	vascular energetic state
endothelial	contractile__metabolic	10	7.61×10^{-4}	0.00231	No	—	—
endothelial	denervation__sensitivity	9	0.00951	0.0164	No	—	—
endothelial	fap__ecm__inflammation	10	0.0012	0.00341	No	—	—

Supplementary Data S2 (continued)

Compartment	Programme	Genes	Time P	Time FDR	Applicable (Yes/No)	Applicable time FDR	Rationale
endothelial	inflammatory__regenerative__reserve	10	0.0108	0.0179	Yes	0.0166	injury-responsive vascular signalling
endothelial	motor__axonal__support	10	0.379	0.407	No	—	—
endothelial	nmj__integrity	10	3.07×10^{-4}	0.00113	No	—	—
endothelial	proteostasis	8	0.0547	0.0718	Yes	0.0689	general stress-control programme
endothelial	schwann__axon__guidance	10	0.199	0.228	No	—	—
endothelial	stromal__vascular__support	10	3.32×10^{-5}	2.43×10^{-4}	Yes	2.08×10^{-4}	vascular identity and support genes
immune	agrin__musk__achr	8	4.00×10^{-6}	5.39×10^{-5}	No	—	—
immune	calcium__mitochondrial__reserve	10	2.63×10^{-4}	0.00105	No	—	—
immune	contractile__metabolic	10	2.52×10^{-5}	2.01×10^{-4}	No	—	—
immune	denervation__sensitivity	9	7.20×10^{-4}	0.00226	No	—	—
immune	fap__ecm__inflammation	10	3.58×10^{-4}	0.00122	Yes	0.00102	ECM-inflammatory signalling including Spp1 and cytokines
immune	inflammatory__regenerative__reserve	10	0.0017	0.00441	Yes	0.00386	immune and regenerative signalling
immune	motor__axonal__support	10	0.176	0.203	No	—	—
immune	nmj__integrity	10	1.30×10^{-5}	1.15×10^{-4}	No	—	—
immune	proteostasis	8	0.774	0.774	Yes	0.774	general stress-control programme
immune	schwann__axon__guidance	10	0.338	0.367	No	—	—
immune	stromal__vascular__support	10	0.0165	0.0269	No	—	—
mural	agrin__musk__achr	8	0.105	0.125	No	—	—
mural	calcium__mitochondrial__reserve	10	5.69×10^{-5}	3.58×10^{-4}	Yes	2.76×10^{-4}	contractile-perivascular energetic state
mural	contractile__metabolic	10	3.61×10^{-4}	0.00122	No	—	—
mural	denervation__sensitivity	9	0.0357	0.0483	No	—	—
mural	fap__ecm__inflammation	10	0.0176	0.0271	No	—	—
mural	inflammatory__regenerative__reserve	10	0.00145	0.00399	Yes	0.00379	injury-responsive perivascular signalling
mural	motor__axonal__support	10	0.67	0.677	No	—	—
mural	nmj__integrity	10	0.0503	0.067	No	—	—
mural	proteostasis	8	0.0191	0.028	Yes	0.0259	general stress-control programme
mural	schwann__axon__guidance	10	0.507	0.531	No	—	—
mural	stromal__vascular__support	10	0.23	0.256	Yes	0.244	perivascular support genes
myogenic	agrin__musk__achr	8	8.69×10^{-6}	8.50×10^{-5}	No	—	—
myogenic	calcium__mitochondrial__reserve	10	0.00193	0.00485	Yes	0.0041	differentiation-associated energetic programme
myogenic	contractile__metabolic	10	0.00258	0.00598	Yes	0.00488	differentiation-associated contractile programme
myogenic	denervation__sensitivity	9	1.26×10^{-8}	5.56×10^{-7}	Yes	2.15×10^{-7}	injury-responsive myogenic programme
myogenic	fap__ecm__inflammation	10	2.35×10^{-4}	9.84×10^{-4}	No	—	—
myogenic	inflammatory__regenerative__reserve	10	2.01×10^{-4}	9.29×10^{-4}	Yes	6.20×10^{-4}	myogenic activation and regenerative genes
myogenic	motor__axonal__support	10	0.00892	0.0157	No	—	—
myogenic	nmj__integrity	10	3.70×10^{-6}	5.39×10^{-5}	No	—	—
myogenic	proteostasis	8	3.67×10^{-5}	2.48×10^{-4}	Yes	2.08×10^{-4}	general stress-control programme
myogenic	schwann__axon__guidance	10	8.72×10^{-4}	0.00256	No	—	—
myogenic	stromal__vascular__support	10	0.0305	0.0419	No	—	—
myonuclei	agrin__musk__achr	8	0.0182	0.0275	Yes	0.0257	postsynaptic receptor-organising programme
myonuclei	calcium__mitochondrial__reserve	10	0.00994	0.0168	Yes	0.0161	myofibre excitation-metabolic programme
myonuclei	contractile__metabolic	10	1.81×10^{-4}	8.84×10^{-4}	Yes	6.15×10^{-4}	myofibre contractile programme
myonuclei	denervation__sensitivity	9	0.0198	0.0285	Yes	0.0259	canonical denervation response

Supplementary Data S2 (continued)

Compartment	Programme	Genes	Time P	Time FDR	Applicable (Yes/No)	Applicable time FDR	Rationale
myonuclei	fap__ecm__inflammation	10	0.00627	0.0123	No	—	—
myonuclei	inflammatory__regenerative__reserve	10	0.0854	0.107	No	—	—
myonuclei	motor__axonal__support	10	0.00851	0.0153	No	—	—
myonuclei	nmj__integrity	10	0.00428	0.00876	Yes	0.00728	postsynaptic and junctional programme
myonuclei	proteostasis	8	0.0805	0.103	Yes	0.0943	myofibre stress-control programme
myonuclei	schwann__axon__guidance	10	0.0175	0.0271	No	—	—
myonuclei	stromal__vascular__support	10	0.00739	0.0138	No	—	—
tenocyte	agrin__musk__achr	8	0.023	0.0326	No	—	—
tenocyte	calcium__mitochondrial__reserve	10	0.0912	0.111	No	—	—
tenocyte	contractile__metabolic	10	5.34×10^{-4}	0.00174	No	—	—
tenocyte	denervation__sensitivity	9	0.103	0.124	No	—	—
tenocyte	fap__ecm__inflammation	10	0.00161	0.0043	Yes	0.00386	connective-tissue ECM programme
tenocyte	inflammatory__regenerative__reserve	10	0.214	0.241	Yes	0.235	injury-responsive connective-tissue signalling
tenocyte	motor__axonal__support	10	0.562	0.582	No	—	—
tenocyte	nmj__integrity	10	0.00409	0.00873	No	—	—
tenocyte	proteostasis	8	0.267	0.293	Yes	0.275	general stress-control programme
tenocyte	schwann__axon__guidance	10	0.599	0.613	No	—	—
tenocyte	stromal__vascular__support	10	0.00417	0.00873	Yes	0.00728	connective-tissue support programme

Restricted age-by-time results

Compartment	Programme	Genes	Estimate	Age-by-time P	Age-by-time FDR	Applicability (Yes/No)	Applicable age-by-time FDR	Rationale
FAP	agrin__musk__achr	8	0.397	0.0947	0.695	No	—	—
FAP	calcium__mitochondrial__reserve	10	-0.17	0.152	0.729	No	—	—
FAP	contractile__metabolic	10	0.011	0.959	0.983	No	—	—
FAP	denervation__sensitivity	9	0.833	0.00325	0.286	No	—	—
FAP	fap__ecm__inflammation	10	-0.108	0.56	0.945	Yes	0.802	core stromal-ECM programme
FAP	inflammatory__regenerative__reserve	10	-0.227	0.457	0.914	Yes	0.749	injury-responsive stromal signalling
FAP	motor__axonal__support	10	-0.535	0.109	0.729	No	—	—
FAP	nmj__integrity	10	0.248	0.253	0.837	No	—	—
FAP	proteostasis	8	0.601	0.0444	0.521	Yes	0.377	general stress-control programme
FAP	schwann__axon__guidance	10	-0.552	0.127	0.729	No	—	—
FAP	stromal__vascular__support	10	0.0664	0.707	0.945	Yes	0.89	stromal and perivascular support genes
Schwann__neural	agrin__musk__achr	8	-0.476	0.174	0.729	No	—	—
Schwann__neural	calcium__mitochondrial__reserve	10	-0.439	0.161	0.729	No	—	—
Schwann__neural	contractile__metabolic	10	-0.112	0.686	0.945	No	—	—

Compartment	Programme	Genes	Estimate	Age-by-time P	Age-by-time FDR	Applicability (Yes/No)	Applicable age-by-time FDR	Rationale
Schwann__neural	denervation__sensitivity	9	0.16	0.656	0.945	No	—	—
Schwann__neural	fap__ecm__inflammation	10	-0.171	0.673	0.945	No	—	—
Schwann__neural	inflammatory__regenerative__reserve	10	-0.931	0.0648	0.634	Yes	0.441	injury-responsive glial signalling
Schwann__neural	motor__axonal__support	10	-0.109	0.798	0.945	Yes	0.913	glial-neural support programme
Schwann__neural	nmj__integrity	10	-0.451	0.199	0.73	No	—	—
Schwann__neural	proteostasis	8	0.0367	0.943	0.983	Yes	0.972	general stress-control programme
Schwann__neural	schwann__axon__guidance	10	0.286	0.302	0.856	Yes	0.733	Schwann and axon-guidance programme
Schwann__neural	stromal__vascular__support	10	-0.0808	0.784	0.945	No	—	—
endothelial	agrin__musk__achr	8	0.127	0.778	0.945	No	—	—
endothelial	calcium__mitochondrial__reserve	10	-0.179	0.166	0.729	Yes	0.629	vascular energetic state
endothelial	contractile__metabolic	10	0.0181	0.941	0.983	No	—	—
endothelial	denervation__sensitivity	9	0.245	0.452	0.914	No	—	—
endothelial	fap__ecm__inflammation	10	0.181	0.634	0.945	No	—	—
endothelial	inflammatory__regenerative__reserve	10	0.16	0.576	0.945	Yes	0.802	injury-responsive vascular signalling
endothelial	motor__axonal__support	10	-0.106	0.675	0.945	No	—	—
endothelial	nmj__integrity	10	0.19	0.646	0.945	No	—	—
endothelial	proteostasis	8	0.179	0.59	0.945	Yes	0.802	general stress-control programme
endothelial	schwann__axon__guidance	10	4.20×10^{-4}	0.998	0.998	No	—	—
endothelial	stromal__vascular__support	10	-0.243	0.365	0.874	Yes	0.749	vascular identity and support genes
immune	agrin__musk__achr	8	0.314	0.187	0.73	No	—	—
immune	calcium__mitochondrial__reserve	10	-0.243	0.0474	0.521	No	—	—
immune	contractile__metabolic	10	0.199	0.26	0.837	No	—	—
immune	denervation__sensitivity	9	0.431	0.0382	0.521	No	—	—
immune	fap__ecm__inflammation	10	-0.462	0.0831	0.695	Yes	0.471	ECM-inflammatory signalling including Spp1 and cytokines
immune	inflammatory__regenerative__reserve	10	0.0472	0.786	0.945	Yes	0.913	immune and regenerative signalling
immune	motor__axonal__support	10	0.0804	0.735	0.945	No	—	—
immune	nmj__integrity	10	0.495	0.0267	0.521	No	—	—
immune	proteostasis	8	0.0435	0.805	0.945	Yes	0.913	general stress-control programme
immune	schwann__axon__guidance	10	0.204	0.331	0.874	No	—	—
immune	stromal__vascular__support	10	-0.0358	0.857	0.955	No	—	—
mural	agrin__musk__achr	8	0.103	0.742	0.945	No	—	—
mural	calcium__mitochondrial__reserve	10	-0.00924	0.972	0.983	Yes	0.972	contractile-perivascular energetic state
mural	contractile__metabolic	10	0.0527	0.788	0.945	No	—	—
mural	denervation__sensitivity	9	-0.0158	0.963	0.983	No	—	—
mural	fap__ecm__inflammation	10	-0.474	0.174	0.729	No	—	—
mural	inflammatory__regenerative__reserve	10	-0.7	0.0382	0.521	Yes	0.377	injury-responsive perivascular signalling

Compartment	Programme	Genes	Estimate	Age-by-time *P*	Age-by-time FDR	Applicability (Yes/No)	Applicable age-by-time FDR	Rationale
mural	motor__axonal__support	10	-0.192	0.515	0.924	No	—	—
mural	nmj__integrity	10	0.0583	0.85	0.955	No	—	—
mural	proteostasis	8	0.489	0.12	0.729	Yes	0.581	general stress-control programme
mural	schwann__axon__guidance	10	-0.272	0.341	0.874	No	—	—
mural	stromal__vascular__support	10	-0.262	0.397	0.896	Yes	0.749	perivascular support genes
myogenic	agrin__musk__achr	8	-0.0822	0.751	0.945	No	—	—
myogenic	calcium__mitochondrial__reserve	10	-0.526	0.158	0.729	Yes	0.629	differentiation-associated energetic programme
myogenic	contractile__metabolic	10	0.205	0.439	0.914	Yes	0.749	differentiation-associated contractile programme
myogenic	denervation__sensitivity	9	0.726	0.00664	0.292	Yes	0.226	injury-responsive myogenic programme
myogenic	fap__ecm__inflammation	10	0.458	0.284	0.837	No	—	—
myogenic	inflammatory__regenerative__reserve	10	-0.335	0.192	0.73	Yes	0.654	myogenic activation and regenerative genes
myogenic	motor__axonal__support	10	-0.287	0.376	0.874	No	—	—
myogenic	nmj__integrity	10	-0.0593	0.844	0.955	No	—	—
myogenic	proteostasis	8	0.229	0.27	0.837	Yes	0.733	general stress-control programme
myogenic	schwann__axon__guidance	10	-0.0527	0.828	0.955	No	—	—
myogenic	stromal__vascular__support	10	0.168	0.737	0.945	No	—	—
myonuclei	agrin__musk__achr	8	0.348	0.377	0.874	Yes	0.749	postsynaptic receptor-organising programme
myonuclei	calcium__mitochondrial__reserve	10	-0.218	0.688	0.945	Yes	0.89	myofibre excitation-metabolic programme
myonuclei	contractile__metabolic	10	-0.0223	0.961	0.983	Yes	0.972	myofibre contractile programme
myonuclei	denervation__sensitivity	9	1.14	0.0134	0.393	Yes	0.228	canonical denervation response
myonuclei	fap__ecm__inflammation	10	-0.267	0.591	0.945	No	—	—
myonuclei	inflammatory__regenerative__reserve	10	-0.0175	0.971	0.983	No	—	—
myonuclei	motor__axonal__support	10	-0.0158	0.377	0.874	No	—	—
myonuclei	nmj__integrity	10	0.417	0.238	0.837	Yes	0.733	postsynaptic and junctional programme
myonuclei	proteostasis	8	0.428	0.485	0.914	Yes	0.749	myofibre stress-control programme
myonuclei	schwann__axon__guidance	10	0.0586	0.688	0.945	No	—	—
myonuclei	stromal__vascular__support	10	-0.303	0.529	0.932	No	—	—
tenocyte	agrin__musk__achr	8	-0.15	0.687	0.945	No	—	—
tenocyte	calcium__mitochondrial__reserve	10	-0.287	0.442	0.914	No	—	—
tenocyte	contractile__metabolic	10	-0.193	0.488	0.914	No	—	—
tenocyte	denervation__sensitivity	9	0.423	0.0878	0.695	No	—	—
tenocyte	fap__ecm__inflammation	10	-0.0465	0.922	0.983	Yes	0.972	connective-tissue ECM programme
tenocyte	inflammatory__regenerative__reserve	10	-0.341	0.463	0.914	Yes	0.749	injury-responsive connective-tissue signalling
tenocyte	motor__axonal__support	10	-0.231	0.56	0.945	No	—	—
tenocyte	nmj__integrity	10	-0.308	0.342	0.874	No	—	—
tenocyte	proteostasis	8	0.254	0.285	0.837	Yes	0.733	general stress-control programme
tenocyte	schwann__axon__guidance	10	-0.268	0.512	0.924	No	—	—
tenocyte	stromal__vascular__support	10	-0.359	0.476	0.914	Yes	0.749	connective-tissue support programme
