# Supplementary material for: Compartment-Specific Transcriptional Remodelling Defines Denervation and Geriatric Muscle Regeneration
Source: Int J Mol Sci. 2026 Oct 12;27(20):9025. doi: 10.3390/ijms27209025 (PMC13705149; doi:10.3390/ijms27209025)
Supplement: Supplementary file 1 [file ijms-27-09025-s001.zip › Supplementary_Figures.pdf]

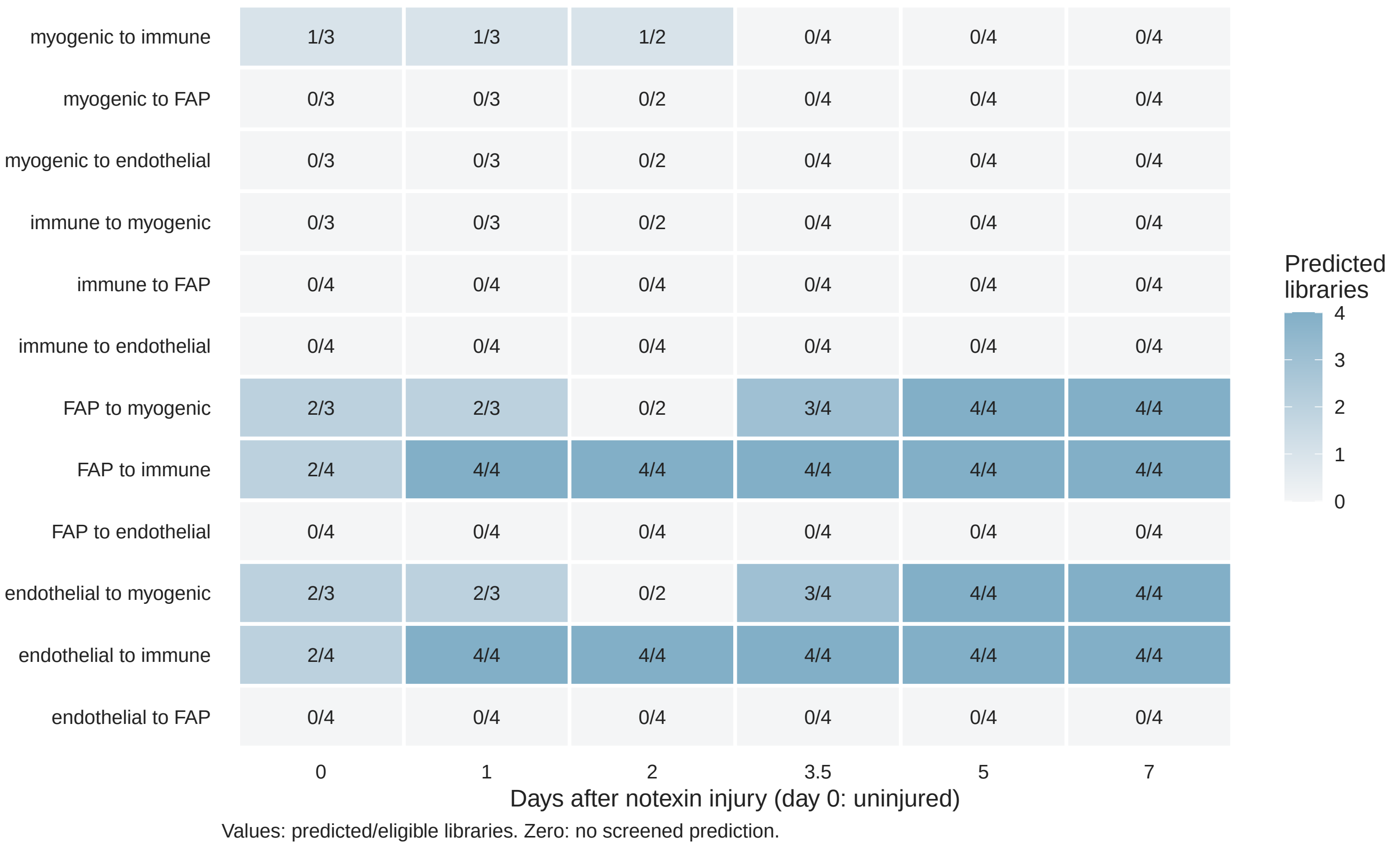

Figure S1. CXCL12-CXCR4 prediction recurrence. All 12 cross-group directions are shown across six geriatric days. Fractions give screened predictions among libraries in which both groups meet the 20-cell threshold (two to four eligible libraries). Tile colour shows the numerator. CellChat screening uses nominal cell-label permutation $P < 0.05$, with 100 permutations per library. Zero means no screened prediction. This is not a test of between-day signalling changes. Source: Supporting Data, Data 8 and Tables 4-5.

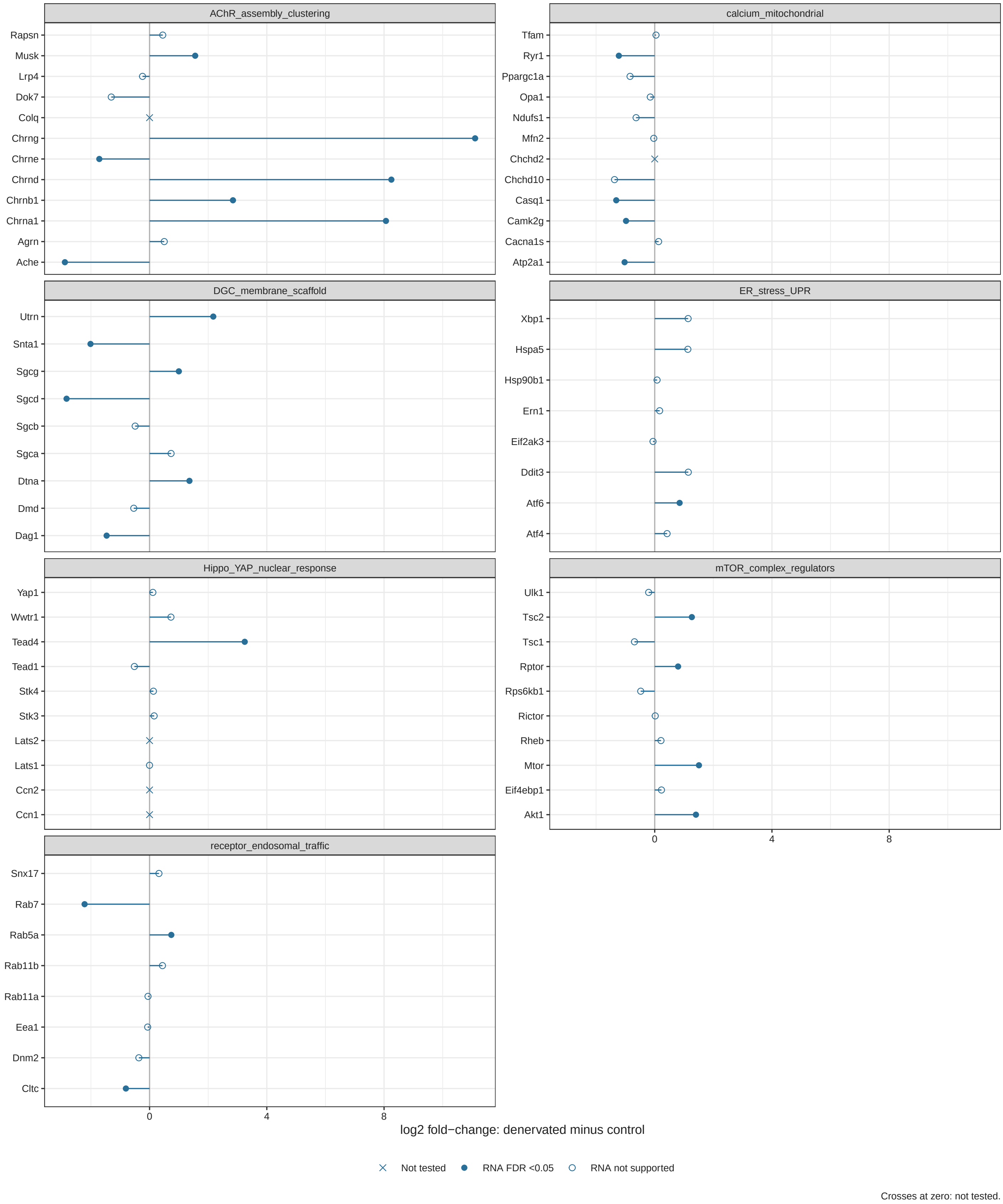

Figure S2. Subcellular-component RNA changes in type IIB nuclei. All genes in seven exploratory panels are shown for GSE183802 animal pseudobulks (three control and three denervated animals). Filled circles: within-cell-type gene-level BH FDR < 0.05; open circles: unsupported tests; crosses at zero: unavailable tests, not measured zero effects. No new panel-level test is assigned. Nuclear RNA does not establish protein localisation, complex assembly, phosphorylation or functional flux. Source: Supporting Data, Data 9.

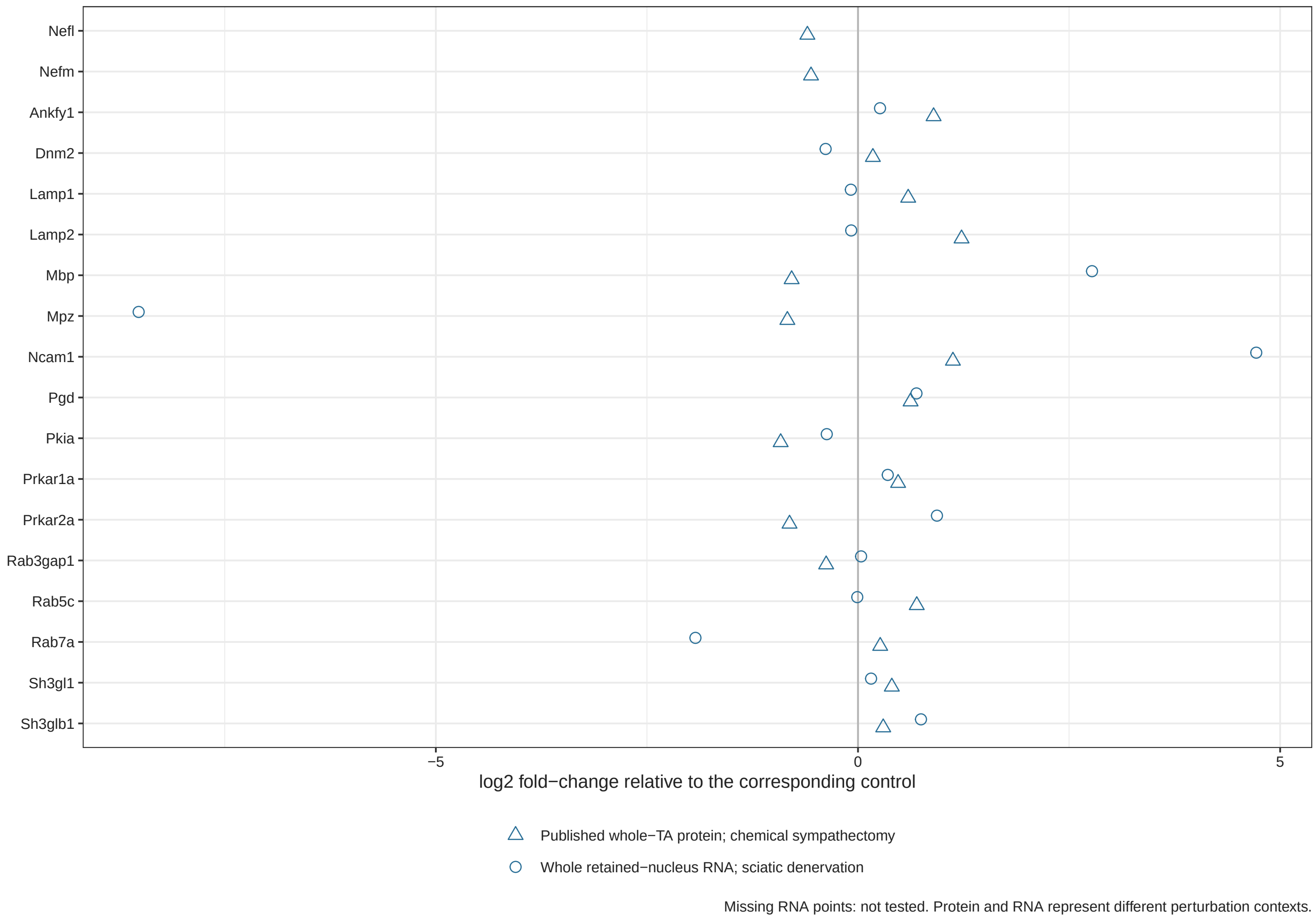

Figure S3. Published trafficking proteins and nuclear RNA. All 18 selected proteins from Straka et al. (2021), Table 2 (doi:10.3389/fphys.2021.626707), are retained. Protein effects are log2-transformed rounded 6OHD/PBS ratios from whole tibialis anterior after two-week chemical sympathectomy (four specimens/group). RNA uses mixed-nucleus GSE183802 animal pseudobulks at day 14 (three animals/group; BH across 12,890 genes). Sixteen RNA tests are available: eight directions agree and eight disagree. Missing RNA points are unavailable tests; Rab7a matches the verified alias Rab7. Shapes identify assays, not significance. Source nominal protein P values are not adjusted abundance tests. This selected, context-mismatched comparison is descriptive, not global validation. Source: Supporting Data, Data 15-16.
